# Characterization and application of LDH with chitosan composites investigated by positron annihilation lifetime spectroscopy and surface texture for the adsorption of methyl orange

**DOI:** 10.1038/s41598-024-65889-2

**Published:** 2024-07-17

**Authors:** E. E. Abdel-Hady, Sarah H. M. Hafez, Hamdy F. M. Mohamed, Mohamed R. M. Elsharkawy

**Affiliations:** 1 Physics Department, Faculty of Science, Minia University, P.O. Box 61519, Minia, Egypt; 2 Physics Department, Higher Institute of Engineering Automotive Technology and Energy in New Heliopolis, Cairo, Egypt

**Keywords:** Methyl orange, Layer double hydroxide, LDH, Adsorption, Zn–Co–Fe, Chitosan, Adsorbent material, Applied physics, Chemical physics

## Abstract

With a rapid increase in industrial growth around the world, the demand for an entirely novel category of nanoparticles and technologies for wastewater treatment has become a key concern for environmental protection. Recently, hybrids of layered double hydroxides (LDH), particularly those containing LDH, have gained attention as potential nanoscale adsorbents for water treatment. Recent research has shown that LDH-containing composites are interesting versatile materials with the ability to be used in energy storage, photocatalysis, nanocomposites, and water treatment. In the current work, LDH-containing composites were utilized as adsorbents for the purpose of purifying water. The adsorbents investigated are Zn–Co–Fe/LDH/Chitosan-in situ sample preparation (LDH/CS1) and Zn–Co–Fe/LDH/Chitosan-ex situ sample preparation (LDH/CS2). Furthermore, LDH/CS1 and LDH/CS2 were investigated for wastewater treatment from methyl orange dye (MO) with various adsorption conditions. When the initial MO concentration was 20 mg/L and the amount of adsorbent was 0.1 g, the removal efficiency reached 72.8 and 91.7% for LDH/CS1 and LDH/CS2, respectively. The MO’s maximum adsorption capabilities are 160.78 and 165.89 mg/g for LDH/CS1 and LDH/CS2, respectively, which is much greater than that of comparable commercial adsorbents. MO adsorption onto LDH/CS1 and LDH/CS2 was best characterized by the pseudo-second-order kinetic model. The equilibrium adsorption data was followed by the Freundlich and Langmuir models. The adsorption is favorable as evidenced by the equilibrium parameter *R*_*L*_ values for MO adsorption onto LDH/CS1 and LDH/CS2, which were 0.227 and 0.144, respectively. Using the free volume distribution method and the positron annihilation lifetime technique, the nanostructure of the materials was examined.

## Introduction

Textiles are one of the world’s most rapidly expanding industries. The fast industrialization has resulted in environmental challenges as in dye pollutants^1^. Natural dyes are less frequently utilized than synthetic dyes as they are more expensive, difficult to apply, have less variation in color, are unstable, and are less harmful to the environment^2^. Since synthetic dyes are multifaceted and made to be resistant to light, biological, and chemical reactions, dyes that are exposed to water are difficult to get rid of. Dye waste involves harmful and dangerous substances that can block sunlight from reaching the aquatic environment, harming biological activities^3^. A common dye in the textile industry is methyl orange (MO). MO, which is common dye used in the dyeing operation, is well known for being extremely poisonous, carcinogenic, and mutagenic^4^. In order to prevent this dye from being released into aquatic organizations, it must be removed from a waste stream. To remove dyes from an aqueous media, particularly methyl orange, many effluent treatment procedures have been applied, including membrane filtration^5^, chemical oxidation^6^, ozonation^7^, biological treatment^8^, ion exchange^9^, coagulation^10^, flocculation, and adsorption^11,12^. Adsorption is one of the most extensively used processing method due to its excellent removing efficacy, inexpensive processing, and simplicity^13^. To adsorb dyes from wastewater, many materials have been evaluated. Some of them include fly ash^14^, rice husks^15^, zeolites^16^, chitosan^17^, activated carbon^18,19^, and clays^20,21^.

Anionic clay layered double hydroxide (LDH) is commonly stated as [M^2+^_1−*x*_M^3+^_*x*_(OH)_2_]^*x*+^[A^*n*−^_*x*/*n*_]^*x*−^·*m*H_2_O, where M^2+^, M^3+^ and A^*n*−^ indicate divalent metal cations, trivalent metal cations, and an interlayer anion^22,23^. LDHs have recently gained popularity because of their utilization in various industries, counting the treatment of water^24,25^. Due to their enormous surface area, interlayer ion exchange, and layered structure, LDHs have been regarded as good adsorbents for wastewater treatment. It is effective, affordable, uncomplicated, easy to use, and unaffected by dangerous ingredients^26^. The addition of various components to the LDH may enhance the functionality of its surface, hence improving adsorption effectiveness for a variety of contaminants^27–29^. In this study, the combination of LDHs supported by chitosan has been recognized as a suitable adsorbent for water pollution removal.

Natural biopolymer chitosan has recently been investigated as a dye removal adsorbent^30–32^. The usage of chitosan in adsorption processes has increased due to its hydrophilic properties, which are also attributed to its biological compatibility, biodegradability, antibacterial powers, non-toxicity, cost-effectiveness, quick adsorption kinetics, and renewable nature^33^. Anionic dyes were successfully adsorbed by chitosan in both single and binary systems, according to Mahmoodi et al.^34^. A range of pollutants can be adsorbed on chitosan by using its amino and hydroxyl groups as coordination and active sites^35^. Conversely, chitosan’s low porosity, low surface area, pH sensitivity, and limited conductivity have restricted its industrial use. To avoid these drawbacks, composites made from chitosan have been developed and used for improved adsorption and to obtain a porous material^36^. The proposed study seeks to advance the development of LDH/chitosan composites with improved adsorption performance, stability, and cost-effectiveness. The free volume and distribution of these porous composites can be evaluated using positron annihilation lifetime spectroscopy (PALS), a nondestructive and highly sensitive approach.

Positron annihilation lifetime spectroscopy (PALS) is a highly sensitive and promising approach for detecting defects in the structure of solids, including nanomaterials. PALS is a nondestructive technique for studying voids and defects in materials^37^. PALS is based on monitoring the time difference between positron implantation in the material and the emission of annihilation radiation. After implantation, the positron becomes thermalized in the material and can freely diffuse through it. The positron can then either immediately annihilate with an electron, converting their masses into two gamma quanta of 511 keV each, or it can bind with an electron and produce positronium (Ps) before annihilation^38^. Meanwhile, surface texture analysis is also important since it gives accurate details about the surface morphology, roughness, and porosity of the LDH/chitosan composite. Understanding the surface properties of the composite material is vital since they directly affect its adsorption capacity and efficiency. This information is useful in determining the availability of adsorption sites and the distribution of active sites.

This work presents a novel approach to utilizing LDH for removing anionic dye from aqueous solutions through an economical approach. Specifically, the investigation focuses on assessing the effectiveness of the Zn–Co–Fe/LDH/Chitosan composite with different preparation methods for the adsorption of MO dye. The objective is to provide clarity on the characterization and application of LDH/chitosan composites using techniques like positron annihilation lifetime spectroscopy (PALS) and surface texture analysis, with the ultimate goal of describing the adsorption of MO dye. PALS serves as a crucial technique in this study due to its ability to provide unique insights into the structural properties and defects within materials. Furthermore, the aim is to elucidate the adsorption mechanisms specific to MO dye. The influences of different parameters on the adsorption procedure were studied separately, including system pH, dye concentration, temperature, and adsorbent dose. The surface and structural features of the adsorbents were examined using scanning electron microscopy (SEM), X-ray diffraction (XRD), and positron annihilation lifetime (PAL) measuring techniques after the addition of Zn–Co–Fe/LDH layers to chitosan. Kinetic curves were examined using pseudo-first order (PFO) and pseudo-second-order (PSO) models. In order to understand the adsorption of the MO dye on Zn–Co–Fe/LDH/Chitosan, the fitting of the adsorption isotherms to the Langmuir and Freundlich models is further explored. The standard values of entropy (ΔS^o^), enthalpy (ΔH^o^), and Gibbs free energy (ΔG^o^) changes were determined.

## Materials and methods

### Materials

Methyl orange (C_14_H_14_N_3_NaO_3_S), the adsorbate utilized in this investigation, was acquired from Oxford Laboratory Reagent in India. LobaChemie, India, provided the iron nitrate [Fe(NO_3_)_3_⋅9H_2_O], cobalt nitrate [Co(NO_3_)_2_⋅6H_2_O], and zinc nitrate [Zn(NO_3_)_2_⋅6H_2_O] adsorbents. The hydrochloric acid and sodium hydroxide were supplied by Chem-lab NV. Glacial acid (CH_3_CO_2_H- MW: 60.05 g/mol) was supplied by Sigma–Aldrich. Sodium carbonate (CNa_2_O_3_- MW: 105.99 g/mol) was supplied by Alfa Aesar. Chitosan was obtained from Sigma–Aldrich and the substances were used without modification, just as they were received.

### Experimental

A simple chemical co-precipitation and hydrothermal process were employed to prepare the LDH/CS1 (Zn–Co–Fe/LDH/Chitosan-in situ sample preparation). Zinc nitrate, cobalt nitrate, and iron nitrate (Zn–Co–Fe (2:2:1 M ratio)) were first dissolved in deionized water (100 mL) and maintained as solution A. Afterwards, 100 mL solution containing 0.3 mol sodium hydroxide and 0.1 mol sodium carbonate was adjusted and named solution B. Solution B was slowly added into solution A until the precipitate was completely blended. To create a homogenous chitosan solution, 1.2 g of dissolved chitosan was then added to a 2% (v/v) glacial acid solution and vigorously stirred for three hours which was then mixed into the abovementioned combination and stirred steadily for 3 h. The resultant combination was then kept at 110 °C for 24 h in an autoclave made of Teflon-coated stainless steel. The finished mixture was then centrifuged, washed with warm water until the pH of the solution was neutral and then dried for 24 h under a vacuum. The dry LDH/CS1 was then ground into a uniform powder^39^.

**Figure 1:**
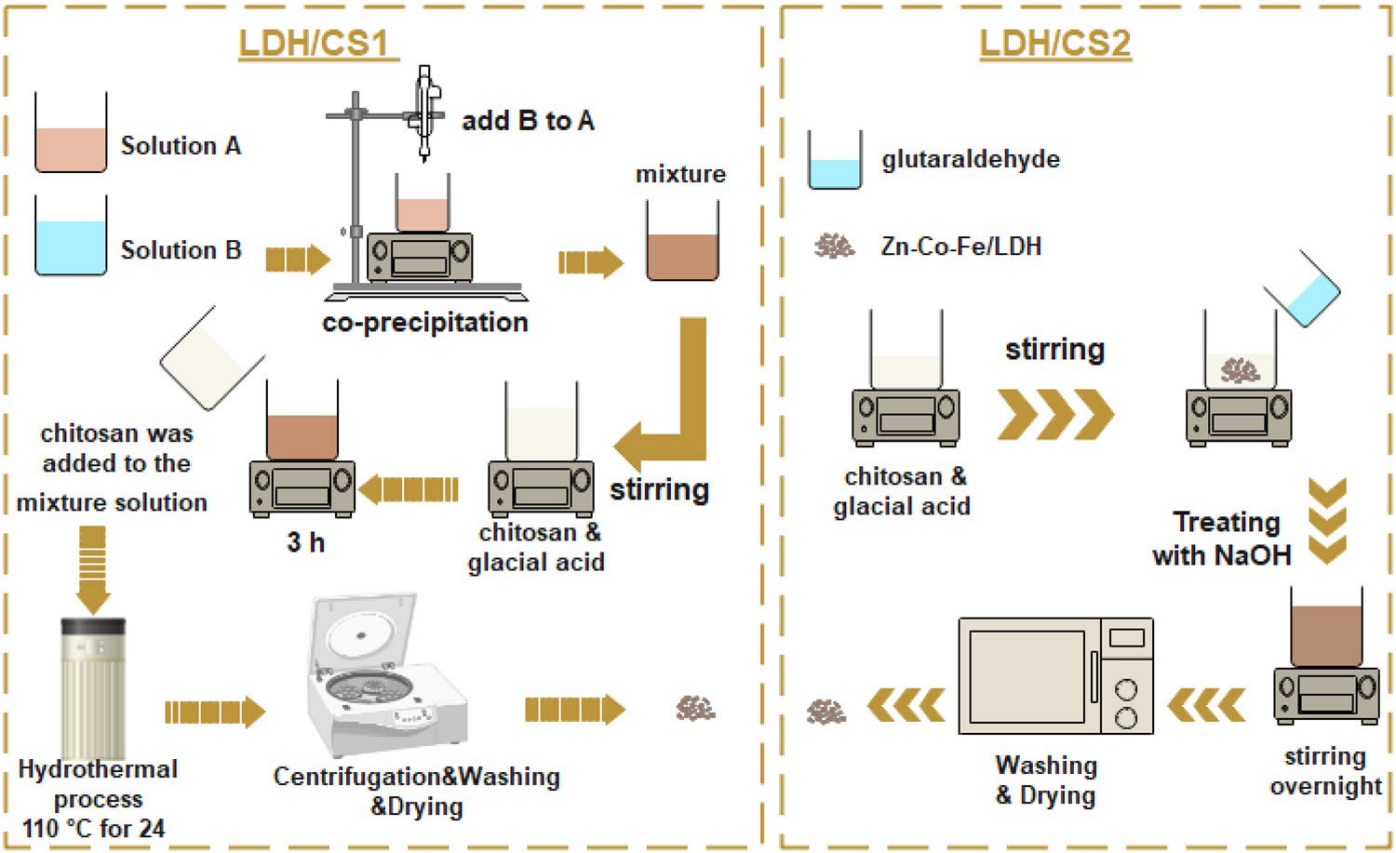
The schematic representations of LDH/CS1 and LDH/CS2.

The Zn–Co–Fe/LDH was prepared using the co-precipitation method as described in our previous work^40^. The zinc nitrate solution was mixed with cobalt and iron nitrate salts and dissolved in 100 mL of deionized water at room temperature. Slowly add a solution of sodium hydroxide (2 mol/L) to the mixture until the precipitate is fully blended and the pH reaches 10. The system was rapidly agitated for another 24 h. The system was then repeatedly filtered and cleaned with distilled water, followed by ethanol, until the pH reached 7. The precipitate was dried in a vacuum oven at 60 °C for 24 h. To prepare LDH/CS2 (Zn–Co–Fe/LDH/Chitosan-ex situ sample preparation), 1 g of Zn–Co–Fe/LDH powder and 10 mL of glutaraldehyde (0.025 M) were simultaneously added to a 100 mL solution of 3 g of chitosan in 2% (v/v) acetic acid and maintained stirring for 6 h. The mixture was treated with NaOH and stirred overnight. The resultant precipitate was cleaned with distilled water and dried in the oven^41^. The schematic representations of LDH/CS1 and LDH/CS2 are shown in Fig. 1.

### Characterization

The morphology was assessed using a JSM-IT200 microscope (Tokyo, Japan) after gold was deposited onto the powdered material on a glass slide using a sputter coater (JEOL-ION SPUTTER JFC-1100). For elemental analysis, energy-dispersive X-ray (EDX) spectroscopy and the sample surfaces were utilized. On a PANalytical (Empyrean), XRD was generated with a Cu Kα incident beam (wavelength of 0.1546 nm, utilizing 2θ = 5–80°) at a tube voltage of 40 kV and a tube current of 30 mA. The measurements were carried out at Minia University in Egypt’s Central Laboratory for Microanalysis and Nanotechnology. With the use of an ultraviolet and visible spectrophotometer (Unico instrument-UV2000-USA), the MO concentration in the samples was determined. A fast–fast coincidence spectrometer was used in a sandwich arrangement to power a ^22^Na positron source for the PAL observations^42^. The PALSfit^43^ software was used to analyse the acquired spectra.

## Result and discussion

### Characterizations

#### XRD

**Figure 2:**
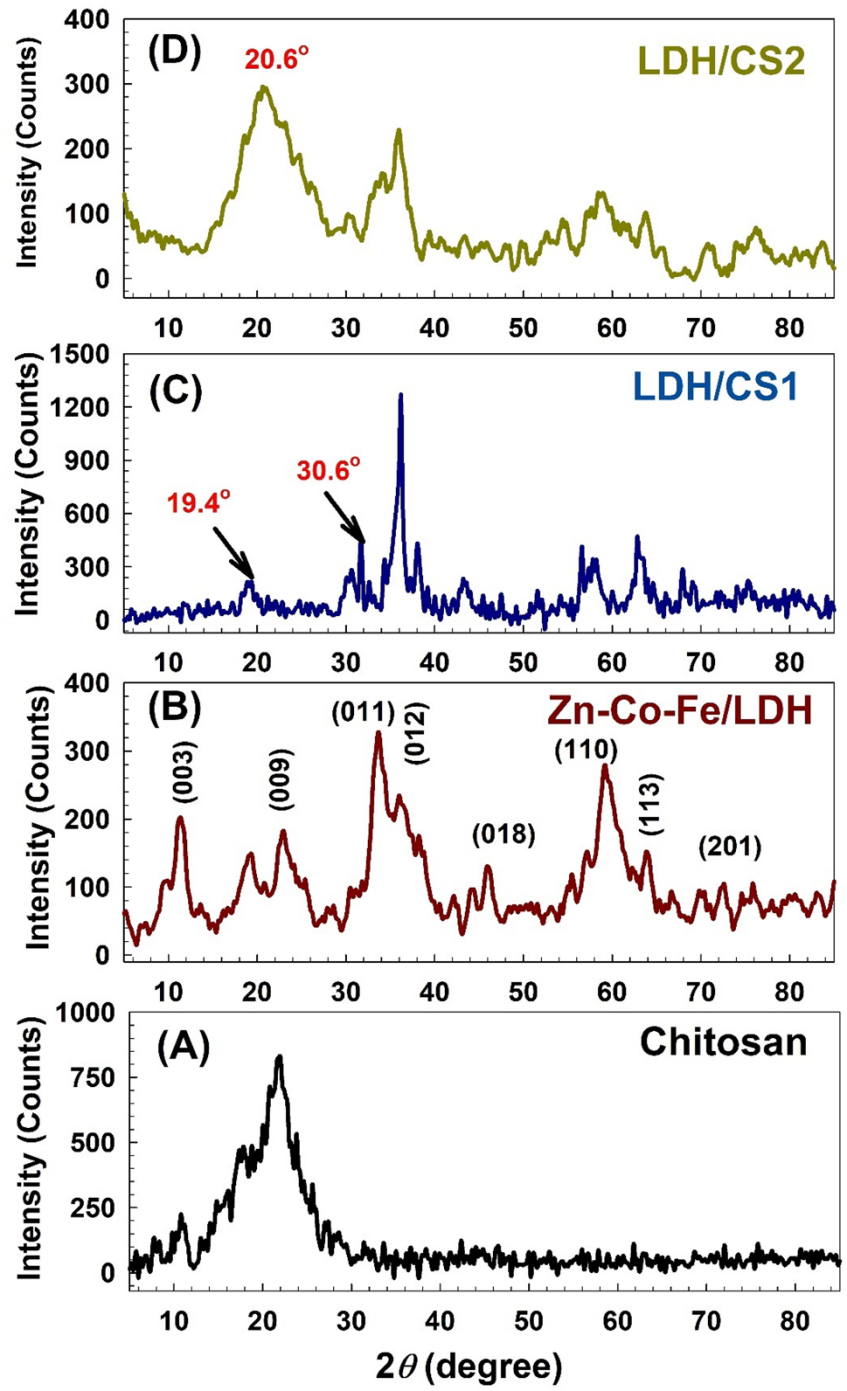
XRD patterns for (**A**) chitosan, (**B**) Zn–Co–Fe/LDH, (**C**) LDH/CS1, and (**D**) LDH/CS2.

The materials’ structure was further characterized using XRD. The prepared chitosan (Fig. 2A) showed a broad reflection at about 2θ = 22° in their XRD patterns. These findings demonstrate the amorphous nature of the chitosan structure^43^. Figure 2B depicts the XRD pattern and shows typical reflections of LDH materials. The main peaks at 2θ of 9.8, 19.4, 33.6, 36.01, 46.00, 59.20, 63.8, and 72.30°, respectively, with reference codes (04–018-3495) and (JC-PDF No. 32–1476), correspond to (003), (009), (011), (012), (018), (110), (113), and (201), respectively. These values confirmed the structure of Zn–Co–Fe/LDH^40^.

Figure 2C displays the XRD pattern for LDH/CS1 and exhibits LDH material reflections and sharpening of peaks. In this pattern, two new peaks appear at 2θ of 19.4° and 30.6°, which are marked in the figure, where the broad reflection peak of chitosan at 2θ = 22° disappeared in the patterns of LDH/CS1. This shows that chitosan is effectively interacting and dispersed between LDH layers^44^. Additionally, the intensity of the reflection of (011) planes increased, and the reflection angle changed from 33.6° in pure LDH to 36.20° in LDH/CS1. According to Sherrer’s formula^45^, the crystalline size will vary from 6.70 to 10.19 nm, and the d-spacing values will change from 2.7 to 2.5 Å for the maximum peak intensity at 2θ of 33.6° in pure LDH, which shifted to the angle 36.20° in LDH/CS1. Figure 2D displays the XRD pattern for the LDH/CS2. It is suggested that the chitosan peaks have shielded the LDH peaks, resulting in the observed broadening in the 2θ = 20.6°^46^. The crystalline size of the LDH/CS2 will be 9.52 nm at an angle of 35.09°. Therefore, in the preparation of LDH/CS2 by using the co-precipitation process to add LDH nanoparticles to chitosan, the crystallinity of the composite was reduced while the flexibility of the polymer chain was increased^47^, compared to the crystalline LDH/CS1, which was prepared using a hydrothermal process.

#### SEM

**Figure 3:**
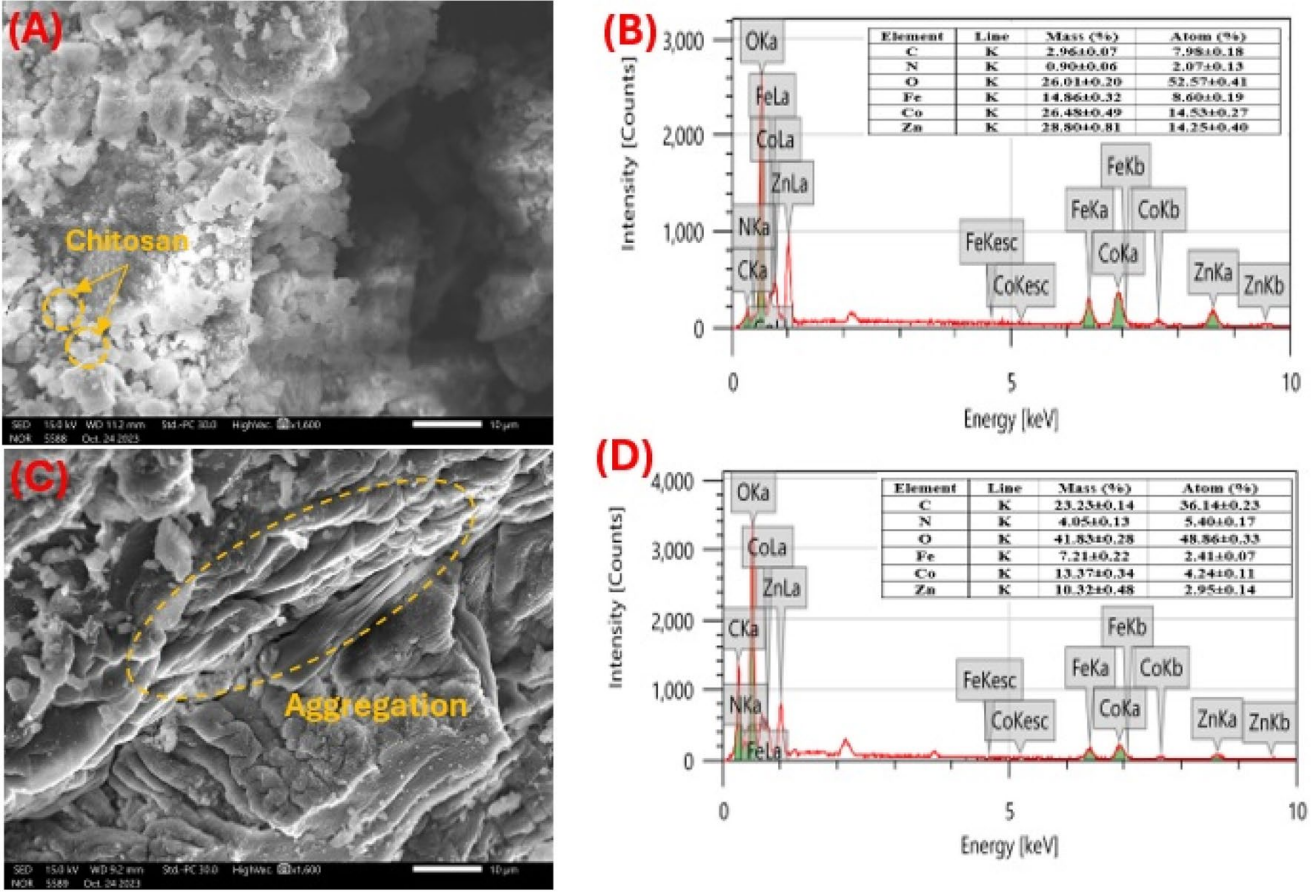
The surface morphology studied by SEM and EDX analysis for (**A**,**B**) LDH/CS1 and (**C**,**D**) LDH/CS2 composites.

The surface morphology of LDH/CS1 and LDH/CS2 composites studied by SEM analysis is presented in Fig. 3. Figure 3A reveals the SEM image of LDH/CS1, from which it was observed that there was a sheet-like shape and irregular agglomerates like sphere pores due to the uniform distribution of the LDH. Furthermore, it was verified that chitosan intercalated into Zn–Co–Fe/LDH in addition to having an excellent dispersion throughout the LDH layers^39^. The LDH/CS2 SEM image is shown in Fig. 3C. Chitosan appears to give LDH compaction, which causes the LDH particles to aggregate. This is caused by interactions between the LDH layers and the surface of the chitosan chains. This could be because of the excellent adhesion that was produced between the layers and chitosan as a result of their strong compatibility^48^. These differences in SEM images reflect the variations in the synthesis mechanisms and reaction conditions associated with the hydrothermal and co-precipitation methods. LDH/CS1 particles synthesized via the hydrothermal method show less agglomeration and better dispersion, with individual particles being more distinct and separated from each other. LDH/CS2 particles prepared by co-precipitation exhibit more agglomeration, with particles tending to aggregate together with fewer surface defects or irregularities due to the slow precipitation process used in the preparation. Additionally, the elemental compositions of the LDH/CS1 and LDH/CS2 were determined by EDX. Figure 3B and D, respectively, illustrates the EDX spectra of the LDH/CS1 and LDH/CS2, which confirmed the presence of metals, oxygen, nitrogen, and carbon. The carbon concentration in LDH/CS1 (7.98 ± 0.18 atom%) is lower than that for LDH/SC2 (36.14 ± 0.23 atom%) which confirm that chitosan is effectively interacting and dispersed between LDH layers as deduced from XRD data.

#### Surface texture and 3D characterization

**Figure 4:**
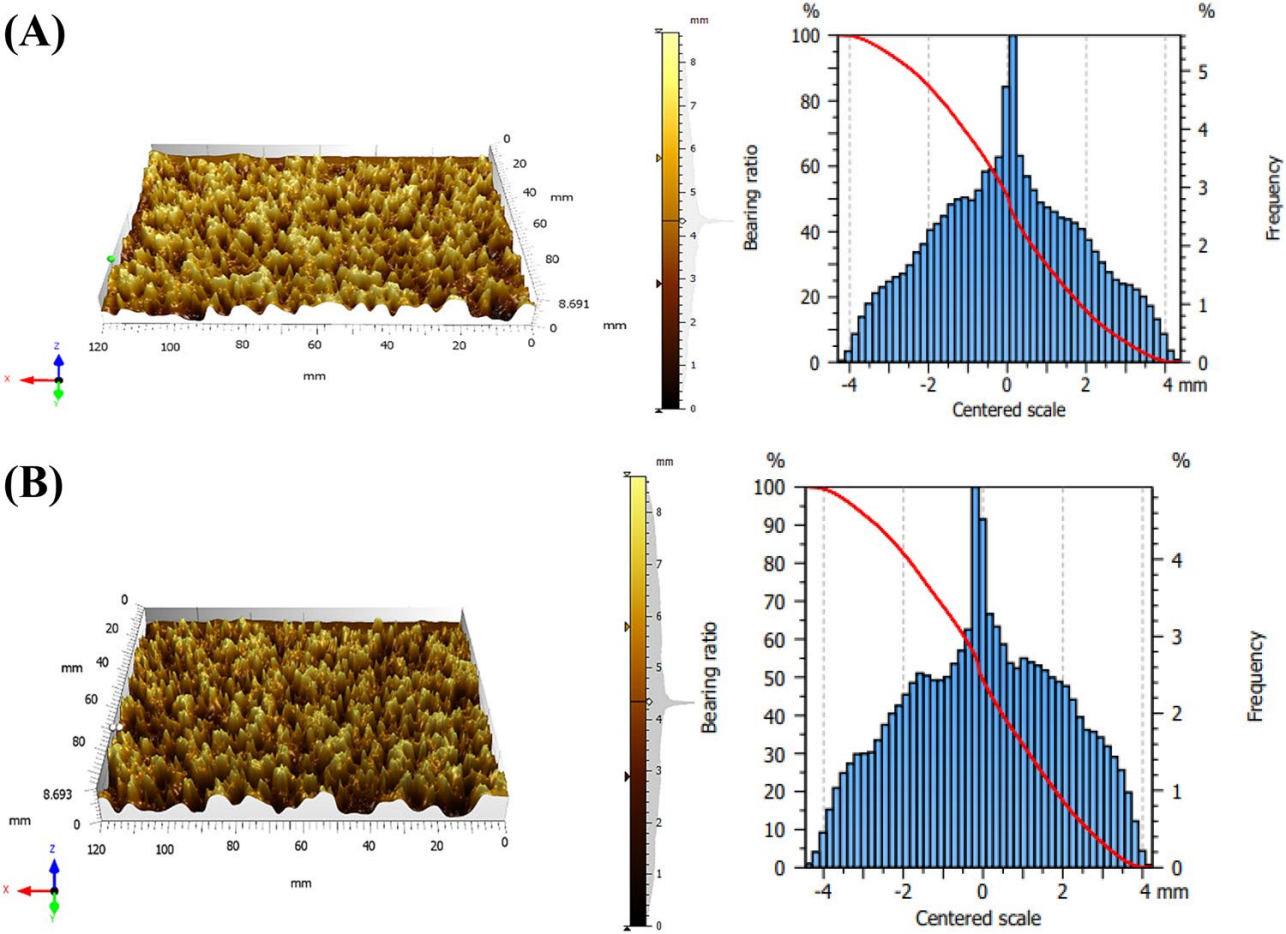
Relevant 3-D SEM micrographs (left figure) and Abbotte Firestone curve and the depth histogram (right figure) for (**A**) LDH/CS1, and (**B**) LDH/CS2.

Surface morphology has garnered a lot of interest since it can reveal essential properties, containing deformations and heterogeneities, that might affect the material’s utilization. Software called Mountain Map^®^ 9.0 was used to analyze the geographical SEM image^49^. Both LDH/CS1 and LDH/CS2 have significant roughness “peaks” and “valleys” visible in their surface profile analyses. Figure 4 displays the Abbott–Firestone curve and sample depth histogram on the right and surface 3D SEM micrographs on the left. The height (or depth) distribution is characterized by a histogram, which shows the probability, or frequency, of points at a given height. The horizontal axis of the Abbott–Firestone curve is graduated in percentages of the overall population, while the vertical axis is graduated in depths. The curve has been colored red.

**Figure 5:**
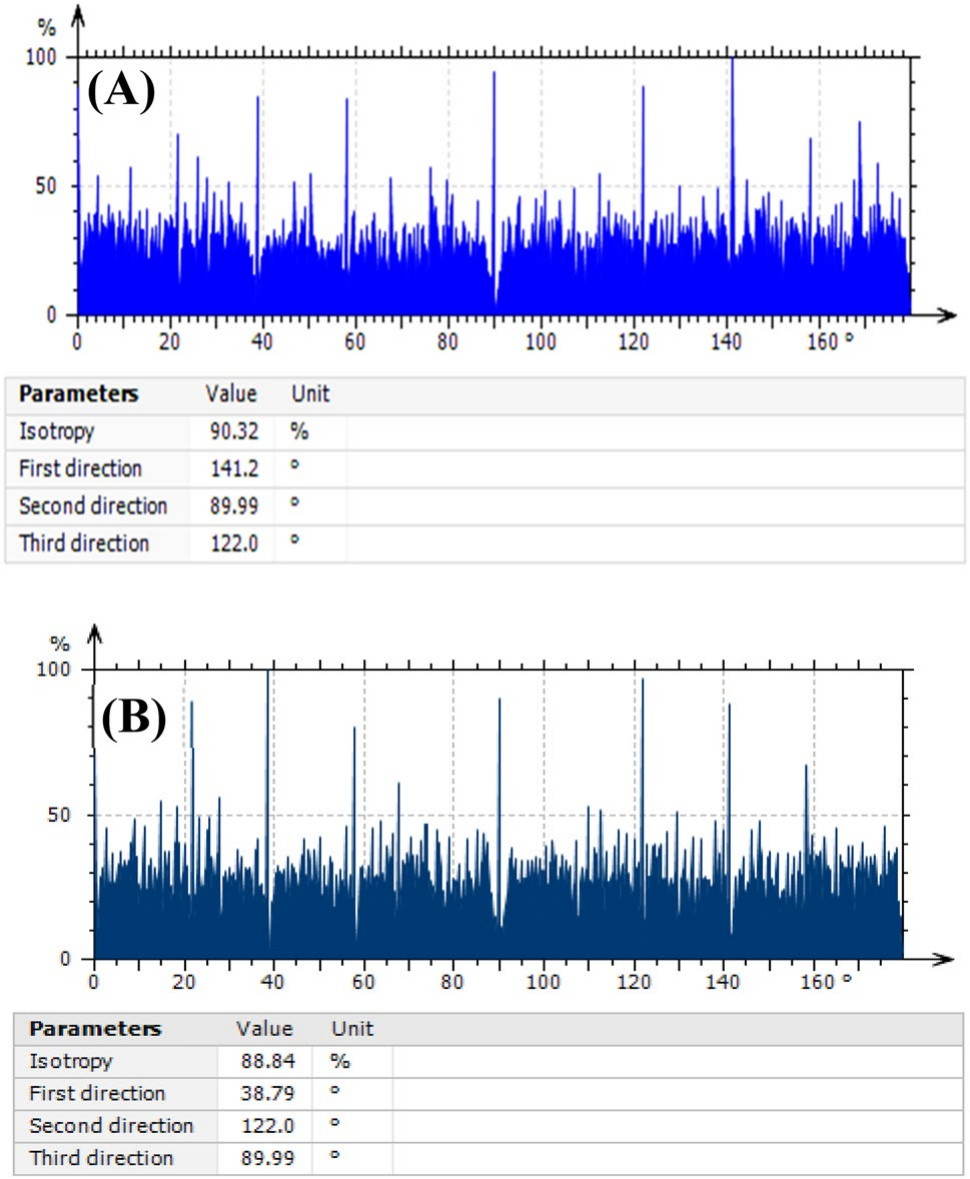
Cartesian graphs of the surface texture directions for (**A**) LDH/CS1, and (**B**) LDH/CS2.

The height distributions of the two samples differ significantly. In addition to the corresponding values of surface texture parameters, which may be connected to surface inhomogeneity, Cartesian graphs (Fig. 5) were utilized to assess the surface texture directions of the samples^50^. The surface parameters are shown in Table 1 in terms of total roughness (Ra), roughness skewness (Rsk), roughness kurtosis (Rku), and fractal dimension (D_f_). The isotropy percentages are 90.32 and 88.84% for LDH/CS1 and LDH/CS2, respectively. This supports the idea that the surface textures of the two samples are isotropic, as indicated by the aspect ratio of the texture (Str) values of 0.8238 and 0.8882 for LDH/CS1 and LDH/CS2, respectively. If Str is near the unit, the surface is isotropic; if Str is near 0 on the other hand, the surface is anisotropic^51^. The kurtosis and skewness features are computed using the square root of the surface height distribution (RMS).

**Table 1:** Textural characteristics of the two samples.

Parameters	Values of parameters	
	LDH/CS1	LDH/CS2
Total roughness (R_t_) (nm)	6.483	5.080
Fractal dimension (D_f_)	1.110	1.161
Slope	0.997	0.999
Roughness skewness (Rsk)	0.3409	− 0.299
Roughness kurtosis (Rku)	3.103	2.438
Void volume (mm^3^/mm^2^)	3.684	3.581

The “sharpness” of a surface and the unpredictability of profile heights are indicated by roughness kurtosis (Rku). Rku = 3 is the value for completely random surfaces, Rku < 3 for bumpy surfaces, and Rku > 3 for spiky surfaces^47^. In this study, Rku values were 3.103 and 2.438 for LDH/CS1 and LDH/CS2, respectively. Because chitosan is present on the LDH surface, LDH/CS1 has a spiky surface, whereas LDH/CS2 has an agglomerated surface that results in a bumpy surface. Roughness skewness (Rsk) is a measure of surface symmetry; a positive number denotes a “peaky” surface, while a negative value indicates that troughs peaky surface^51^. LDH/CS1 has a positive Rsk, which indicates that its surface is peaky. Meanwhile, LDH/CS2 has a negative value, indicating that LDH/CS2’s surface is a valley surface. The void volume of LDH/CS1 is 3.684 mm^3^/mm^2^, while that of LDH/CS2 is 3.581 mm^3^/mm^2^. The decrease in volume is due to agglomeration on the LDH/CS2 surface. The fractional dimension (D_f_) of a sample quantifies its complexity. In this study, the correlation coefficient (R^2^) of the linear fit determined by the enclosing boxes method was close to one, indicating that linear functions matched the data well. As shown in Table 1, the values of D_f_ are 1.110 and 1.161 for LDH/CS1 and LDH/CS2, respectively. The increase in Df reflects a decrease in the production of more regular structures^52^.

#### The positron annihilation lifetime (PAL) studies

When positrons become strongly localized, positronium, a metastable bound state, can arise in low electron density places. When the ortho-positronium (*o*-Ps) spin singlet state is formed, the positron and electron spin alignment is parallel, and it becomes more prevalent than the para-positronium (*p*-Ps) spin singlet state, where the positron and electron spin alignment is anti-parallel. As a matter of fact, the two states have a formation ratio of 1:3. As a result, a little para-positronium contribution with a lifetime of 0.125 ns will also exist in τ_1_. The third lifetime component (τ_3_, *I*_*3*_) has the longest lifetime with respect to the creation and subsequent annihilation of *o*-Ps atoms, a metastable spin-triplet-bound state of the electron and positron (where both spins are parallel). In the current samples, the PAL spectra are best represented by three lifetime components with a variance ratio of less than 1.2, as determined by the PALSfit program^45^. Table 2 presents the three positron lifetime components, τ_1_, τ_2_, and τ_3_, along with their corresponding intensities *I*_*1*_*, I*_*2*_*,* and *I*_*3*_ for LDH/CS1 and LDH/CS2. It is widely accepted that the longest component (τ_3_) is attributed to the annihilation of *o*-Ps atoms formed in large pores within materials^53,54^.

**Table 2:** The three positron annihilation lifetime components and their relative intensities for LDH/CS1 and LDH/CS2.

Sample	τ_1_ (ns)	τ_2_ (ns)	τ_3_ (ns)	*I*_*1*_ (%)	*I*_*2*_ (%)	*I*_*3*_ (%)
LDH/CS1	0.125 ± 0.00	0.477 ± 0.002	2.8593 ± 0.050	30.00 ± 0.57	62.50 ± 0.54	8.30 ± 0.17
LDH/CS2	0.125 ± 0.00	0.467 ± 0.057	1.7250 ± 0.068	29.65 ± 0.80	61.00 ± 0.75	9.30 ± 0.02

In the experimental PAL data y(t), the resolution function R(t) of the PAL system, and a continuous distribution are expressed as the symbol * in a convoluted manner as^55^;

1 $$y\left( t \right) = R\left( t \right)*\left[ {N_{t} \mathop \smallint \limits_{0}^{\infty } \left( {I\left( \tau \right)/\tau } \right)\exp ( - t/\tau )d\tau + B} \right] ,$$

where

2 $$\mathop \smallint \limits_{0}^{\infty } I\left( \tau \right)d\tau = 1,$$

B is the background and *N*_t_ is the total count of the PAL spectrum. The conventional discrete term analysis utilized the LT10.0 program^56–59^ to fit experimental PAL data points to the model function Eq. (1), which contains the number of terms in the spectrum. The annihilation rates λ of positronium in vacancies, equaling 1/τ_3_, take into account the normal distribution using the LT10.0 program.

3 $$\alpha_{3} \left( {\uplambda } \right) = - \left( {2\pi } \right)^{ - 0.5} \sigma_{3}^{ - 1} exp\left\{ { - \frac{{\left[ {Ln\left( {\uplambda } \right) - Ln\left( {{\uplambda }_{30} } \right)} \right]^{2} }}{{2\sigma_{3}^{2} }}} \right\}{\uplambda }^{ - 1} d{\uplambda }.$$

The lifetime distribution of *o*-Ps, denoted as α_3_(τ_3_), is determined by the dispersion of the distribution σ_3_ and the natural logarithm Ln as

4 $$\alpha_{3} \left( {{\uptau }_{3} } \right) = - \left( {2\pi } \right)^{ - 0.5} \sigma_{3}^{ - 1} exp\left\{ { - \frac{{\left[ {Ln\left( {\uplambda } \right) - Ln\left( {{\uplambda }_{30} } \right)} \right]^{2} }}{{2\sigma_{3}^{2} }}} \right\}{\uplambda }^{ - 1} d\lambda\frac{{d{\uptau }_{3} }}{{d{\uplambda }}} = - \left( {2\pi } \right)^{ - 0.5} \sigma_{3}^{ - 1} exp\left\{ { - \frac{{\left[ {Ln\left( {\uplambda } \right) - Ln\left( {{\uplambda }_{30} } \right)} \right]^{2} }}{{2\sigma_{3}^{2} }}} \right\}{\uplambda }^{ - 1} d{\uptau }_{3} .$$

The peak annihilation rate of positronium in vacancies, denoted as λ_30_, provides quantitative evidence for the vacancies or free volume size from the PAL spectrum, as explained by the positronium hole theory^60^. The *o*-Ps lifetime (τ_3_) was utilized to explore the average radius (*R*) of the vacancies, determined using the relationship presented by Tao^60^ and Eldrup et al.^61^ as

5 $$\tau_{3} = 0.5\left\{ {1 - \frac{R}{{R_{o} }} + \frac{1}{2\pi }sin\frac{2\pi R}{{R_{o} }}} \right\}^{ - 1} \,\left( {ns} \right),$$

The equation *R*_*o*_ equals *R* plus the change in *R*, where the change in *R* is 0.1656 nm, represents the thickness of the homogeneous electron layer in which the positron annihilates, as indicated in Nakanishi^59^. The size of the vacancies *V*, measured in nm^3^, is provided as;

6 $$V\, = \,4\pi R^{3} /3.$$

So that the vacancy size distribution is expressed as^26^;

7 $$\alpha_{3} \left( V \right) = - \left( {2\pi } \right)^{ - 0.5} \sigma_{3}^{ - 1} exp\left\{ { - \frac{{\left[ {Ln\left( {\uplambda } \right) - Ln\left( {{\uplambda }_{30} } \right)} \right]^{2} }}{{2\sigma_{3}^{2} }}} \right\}{\uplambda }^{ - 1} d{\uplambda }\frac{dV}{{d{\uplambda }}},$$

where

8 $$\frac{dV}{d\lambda} = - \frac{{4\pi R^{2} \left( {R + R_{o} } \right)^{2} }}{{2R_{o} }}\left\{ {1 - cos\frac{2\pi R}{{R + R_{o} }}} \right\}^{ - 1} ,$$

as derived from Eqs. (5), (6).

**Figure 6:**
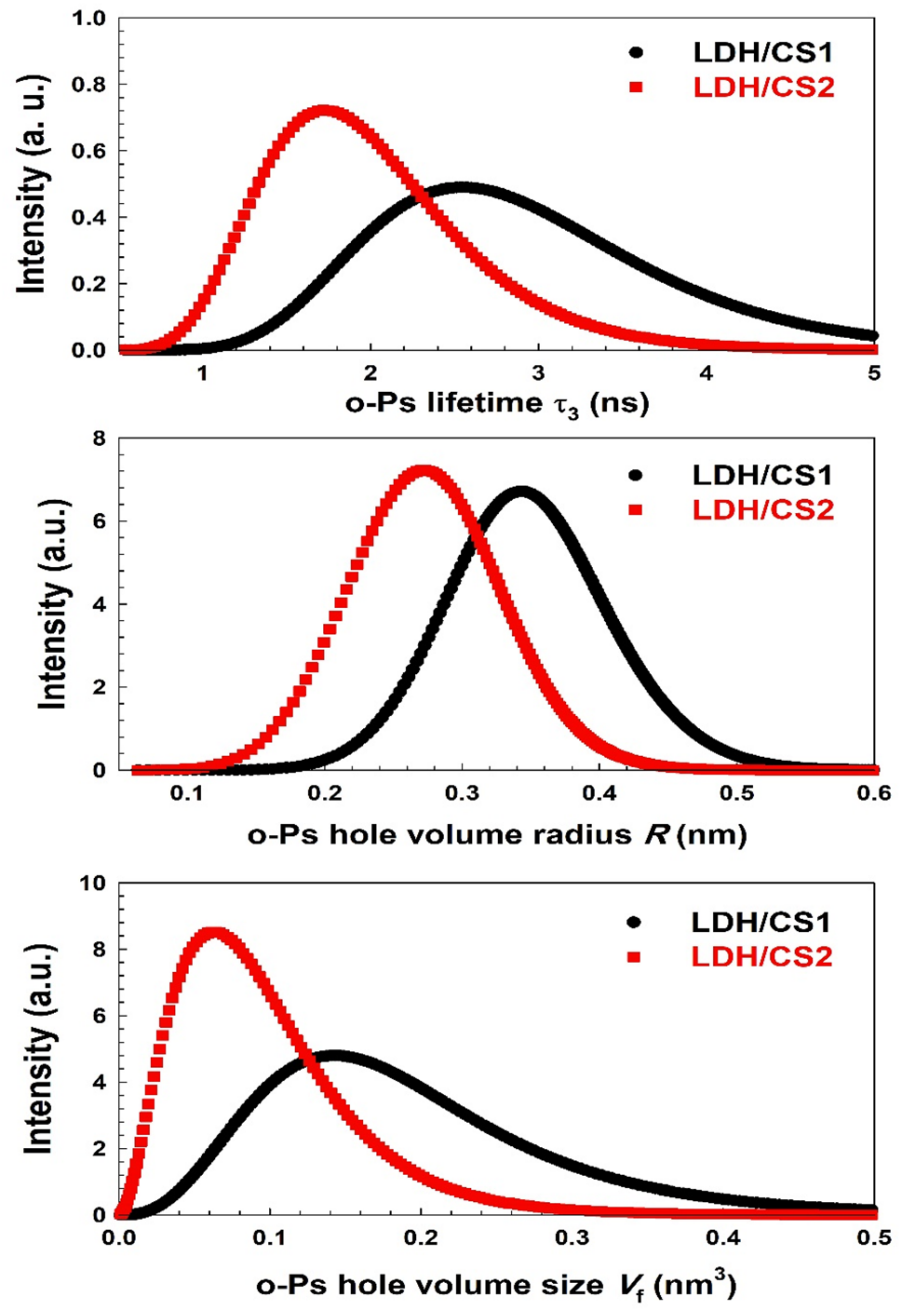
The lifetime, hole volume radius, and free volume distributions for LDH/CS1 and LDH/CS2.

As listed in Table 2, the value of *I*_*3*_ increased in LDH/CS2 as compared to LDH/CS1. The increasing value of *I*_*3*_ in LDH/CS2 indicates that the number density of pores increased, resulting in a decrease in the values of τ_3_, hole radius and *V* from the value in LDH/CS2. In the LDH/CS2 sample, the number of holes increased, but the size of each hole decreased. Since I_3_’s value and sample crystallinity are related^45^, a decrease in LDH/CS2’s crystallinity can be linked to an increase in *I*_*3*_ (Fig. 2D). Figure 6 depicts the *o*-Ps lifetime τ_3_, *o*-Ps hole volume radius *R*, and *o*-Ps hole volume size *V* distributions of the samples. The *o*-Ps hole volume size was calculated using the *o*-Ps lifetime^62,63^. According to PAL analysis, the *o*-Ps lifetime distribution was a typical Gaussian type^64^. The figure shows that the *o*-Ps hole volume size gradually decreases in the LDH/CS2. The o-Ps lifetime exhibited comparable behavior to the *o*-Ps hole volume radius *R*. Because the LDH/CS1 was manufactured using a hydrothermal technique, the LDH/CS1 hole expanded to larger sizes, which is why the hole size distribution differed between the two samples. Meanwhile, LDH/CS2 was synthesized using the co-precipitation process.

**Table 3:** Parameters calculated by Langmuir and Freundlich models for MO adsorption on LDH/CS1 and LDH/CS2.

Isotherm models	Parameters	LDH/CS1	LDH/CS2
Langmuir	q_max_ (mg/g)	160.78	165.89
	*K*_*L*_ (L/mg)	0.0034	0.0059
	R^2^	0.977	0.987
	*R*_*L*_	0.227	0.144
Freundlich	*K*_*F*_ (mg/g)/(mg/L)^1/n^	1.829	3.877
	1/*n*	0.670	0.585
	R^2^	0.991	0.997

### Adsorption studies

In order to investigate the effects of several parameters on the removal of MO from an aqueous solution, batch adsorption experiments were conducted. These parameters included dosage, initial concentration, pH, contact time, and temperature. Each batch experiment was prepared by mixing a stock solution of MO with a concentration of 20 mg/L. These factors include changing the pH of this solution (from 3 to 9) using 0.1 M NaOH and 0.1 M HCl using a pH meter, different time intervals (5–150 min), the influence of dose adsorbent (0.01–0.3 g), and the effect of the initial concentration of MO (10–1000) at 300 rpm on an orbital shaker over 24 h at room temperature. Following the collection of nanoadsorbent, the concentration of MO in the wastewater was calculated. After filtration of the adsorbent by centrifugation, the quantities of MO were measured using a UV–visible spectrophotometer. The efficiency of the adsorption process was estimated using the dye removal efficiency *RE* (%) and adsorption capacity q_t_ (mg/g) as follows^65,66^:

9 $$RE\left( \% \right) = \frac{{C_{o} - C_{t} }}{{C_{o} }}*100$$

10 $$q_{t} = \frac{{C_{o} - C_{t} }}{m} V$$

The weight of the adsorbent (g) is denoted by m, the volume of the solution (L) by V, and the dye concentrations at time 0 and t, respectively, are given by C_o_ and C_t_ (mg/L)^67^.

#### Impact of pH

**Figure 7:**
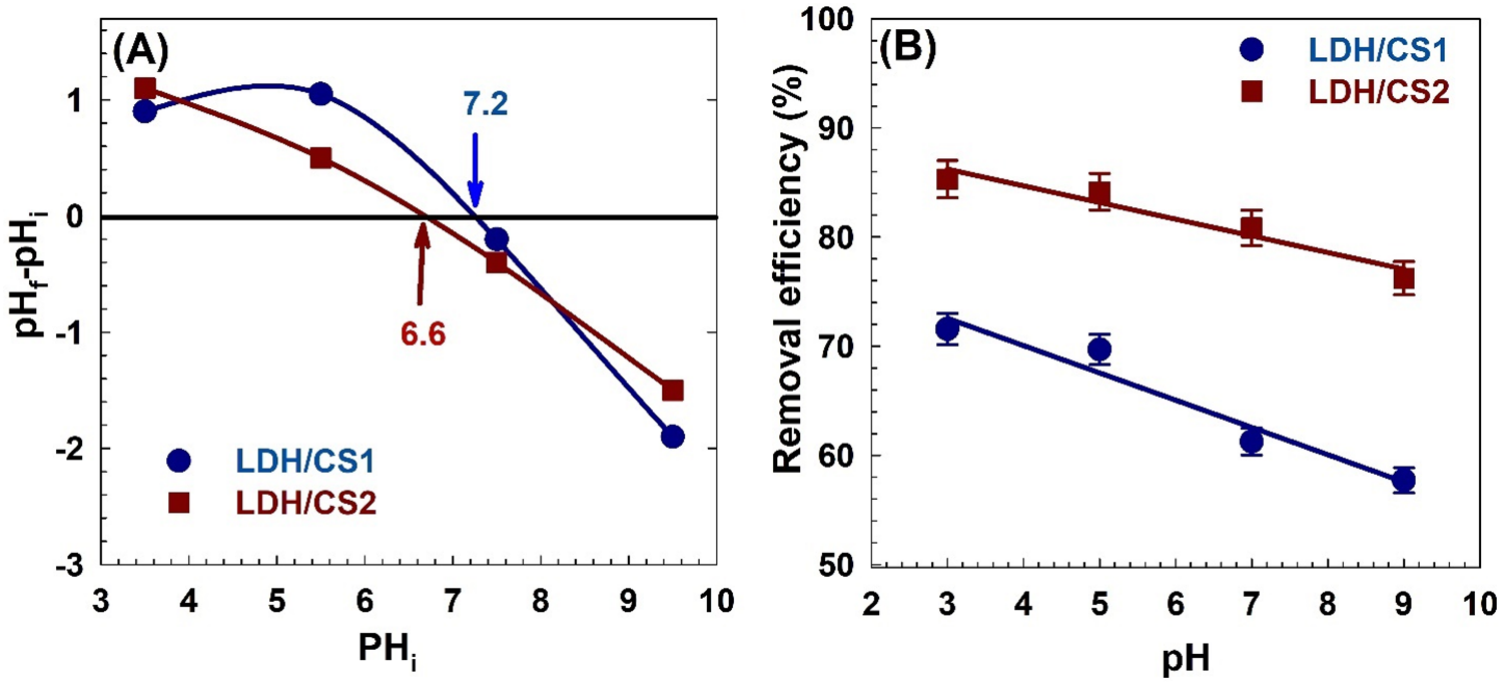
(**A**) point of zero charge of LDH/CS1 and LDH/CS2. (**B**) Effect of pH soluion on the adsorption of MO.

The removal effectiveness of MO dye that was adsorbed into LDH/CS1 and LDH/CS2 from its aqueous suspension at various pH levels was studied with a constant dosage of adsorbent (0.07 g). At 25 °C for 120 min, the adsorbent nanoparticles’ capacity for adsorption in a 20 ppm MO dye solution at various pH levels was determined. Generally, LDH/CS1 and LDH/CS2 showed a larger capacity for MO dye adsorption in acidic medium and a significantly decreased capacity in alkaline pH. Electrostatic repulsion is most likely the cause of the decrease in MO adsorption at alkaline pH. Figure 7A shows the point of zero charge of LDH/CS1 and LDH/CS2. As can be shown in the figure, the adsorption capacity of LDH/CS1 and LDH/CS2 was dictated by their respective pH at the point of zero charge (pzc), which was 7.2 and 6.6. When the pH was above the pzc value, the negative surface charge repelled the incoming flow of the anionic MO dye to prevent and reduce MO adsorption on the adsorbents.

Figure 7B demonstrates the influence of pH on the removal of dyes. LDH/CS1 and LDH/CS2 nanoparticles’ positively charged surfaces attracted anionic dye into their positively charged interlayer region to a greater extent in the pH range of 3–5. It demonstrated the largest level of adsorption on pH 3 for LDH/CS1 and LDH/CS2, and the removal efficiency reached 71.5 and 85.3%, respectively. The removal efficiency decreased at pH 5 due to the domination of H ions in the solution, which generated a competition between the LDH-positive surface and the solution containing excessive H ions, which decreased the adsorption. Meanwhile, the adsorptivity of LDH/CS1 on MO dye decreased from 61.2 to 57% as the MO dye solution’s pH was further increased from 7 to 9. The removal efficiency of LDH/CS2 on MO dye decreased from 80.8 to 76.2% as the MO dye solution’s pH was increased from 7 to 9, respectively.

#### Impact of adsorbent dosage

**Figure 8:**
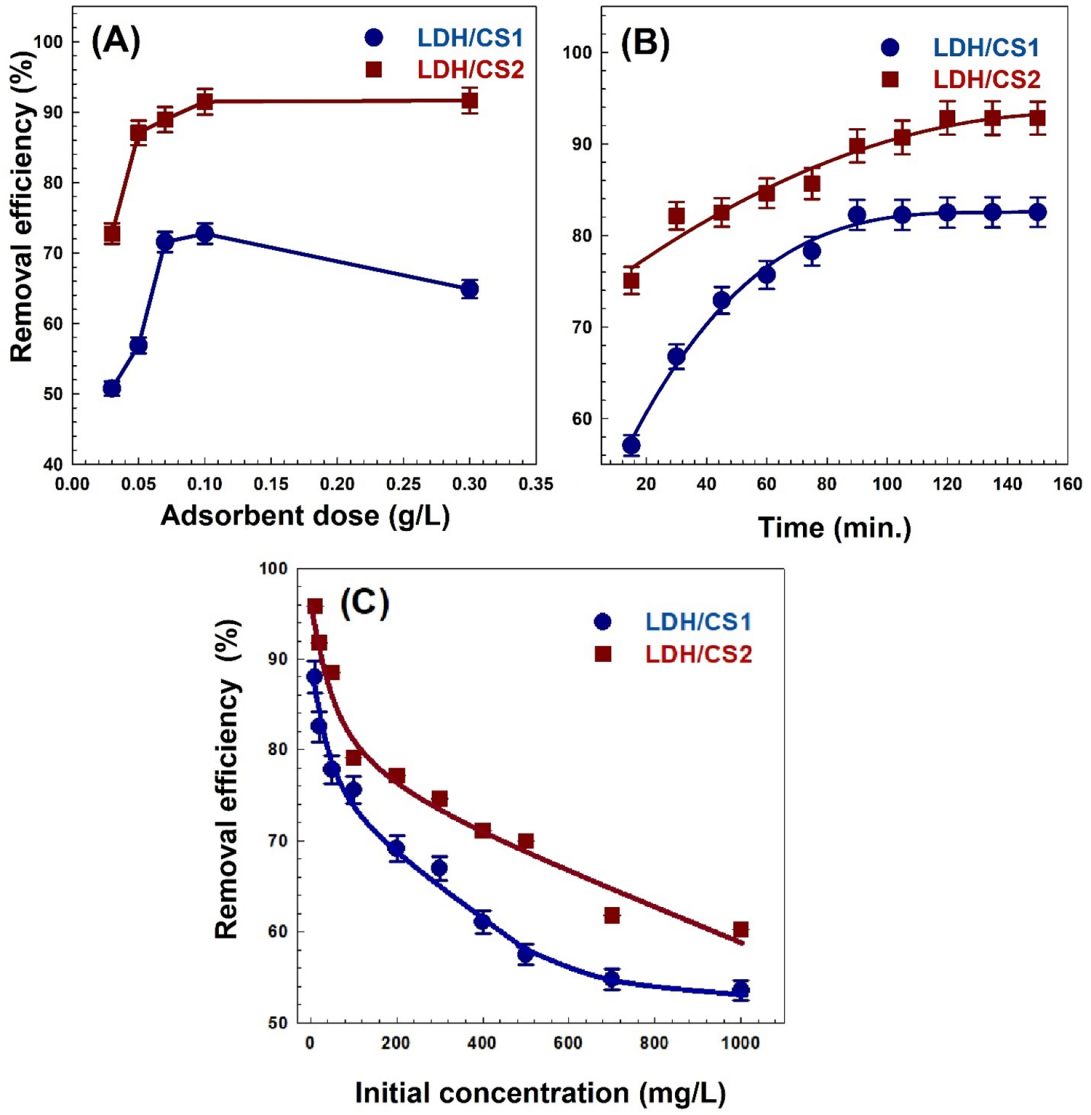
The effect of (**A**) adsorbent dose, (**B**) contact time, and (**C**) concentration of MO dye of LDH/CS1and LDH/CS2.

The influence of adsorbent dose on dye removal studies was studied by varying the adsorbent dose from 0.03 to 0.3 g at an optimal pH as presented in Fig. 8A. According to the results, the MO removal efficiency increases steadily with an increasing amount of LDH/CS1 until the amount of adsorbent reaches 0.1 g (72.7% removal). But after an adsorbent dose of 0.1 g, there was a decrease in removal efficiency (63.3%) due to particle aggregation with increasing mass. On the other hand, when the dose of LDH/CS2 was raised from 0.03 to 0.1 g, the MO adsorptivity raised from 72.8 to 91.5%. However, no improvement in removal efficiency was evident after an 0.1 g adsorbent dosage, suggesting that the saturation level was reached due to the lack of dye molecules that could be adsorbed at higher doses^67^.

#### Impact of contact time

The removal effectiveness of MO dye that was adsorbed into LDH/CS1 and LDH/CS2 from its aqueous suspension at various contact times was investigated with a constant dosage of adsorbent 0.1 g and at 25 °C. The equilibrium time required for the adsorption was calculated by evaluating the adsorbent. Adsorptions are dependent on the contact times of the adsorbate. The adsorptivity of MO at a concentration of 20 mg/L was determined for LDH/CS1 and LDH/CS2 as a function of the contact time, which varied from 15 to 150 min, as illustrated in Fig. 8B. Due to the amount of readily available active, the rate of dye adsorption on LDH/CS1 and LDH/CS2 started out quickly but reached equilibrium at 100 min since there were no molecules of dye remaining to adsorb. Clearly, LDH/CS2 showed improved MO adsorption, reaching a removal efficiency of 91.7% compared to LDH/CS1. The LDH/CS1 sample reaches saturation before the LDH/CS2 sample, due to the availability of large pores in the LDH/CS1, which accelerates the adsorption process and intra-particle diffusion.

#### Effect of dye concentration

A key factor in adsorption is the concentration of MO dye, which is an essential indicator when evaluating the effectiveness of adsorbents and determining the maximum concentration for the removal at an ideal dose (0.1 g) and pH (3). As shown in Fig. 8C, the MO concentration range of 10–1000 mg/L has been studied. With a rise in MO concentrations from 10 to 1000 mg/L, the MO adsorptivity of the LDH/CS1 gradually decreased from 87.9 to 53.6%. Meanwhile, with a dye concentration increase from 10 to 1000 mg/L, LDH/CS2 demonstrated a drop in its dye removal from 95.8 to 60.2%. The adsorptivity of the dye falls as concentration increases because of the lack of active sites on the LDH/CS1 and LDH/CS2 surfaces.

### Adsorption isotherms

**Figure 9:**
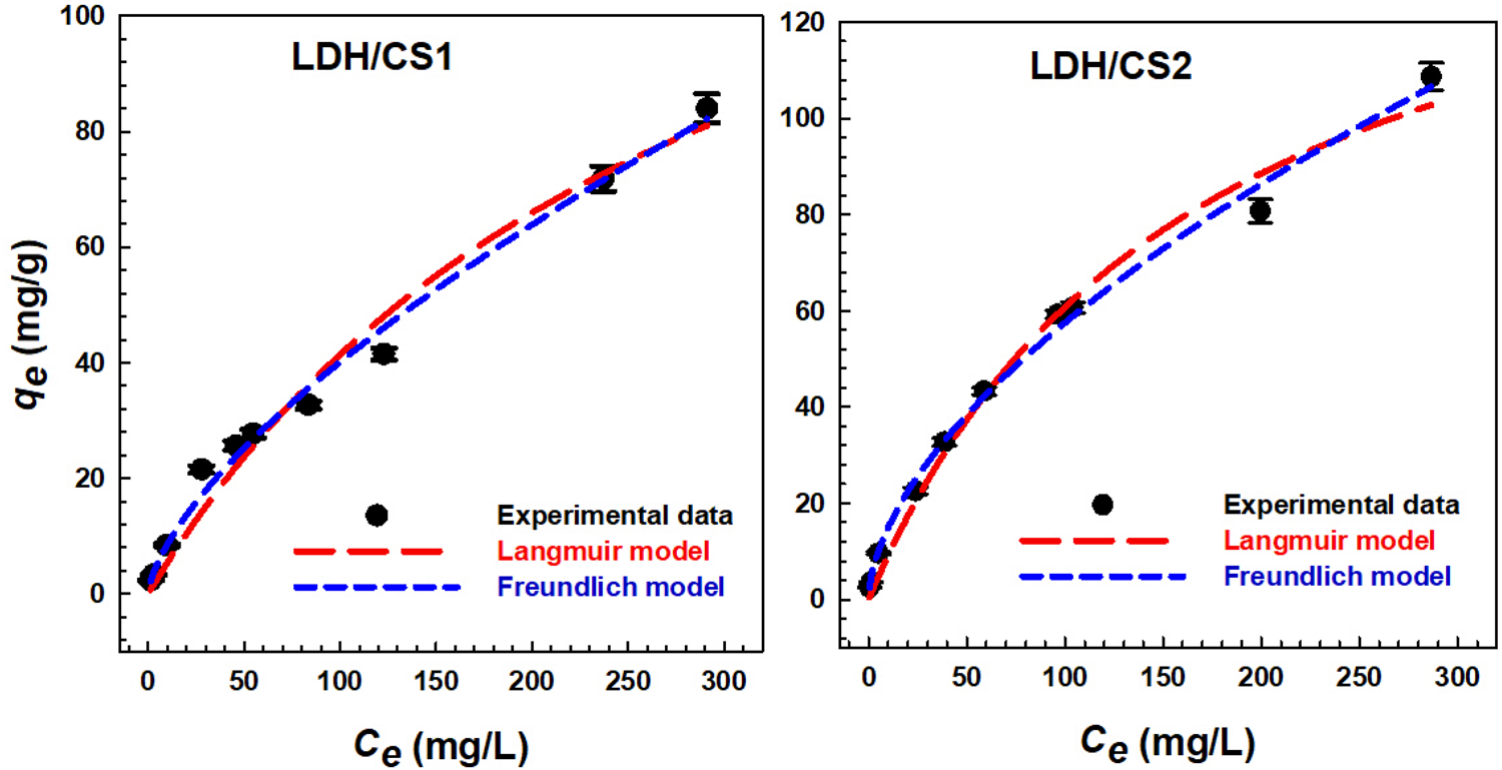
Adsorption isotherms of MO on LDH/CS1 and LDH/CS2 samples.

The adsorption isotherms demonstrate a connection between the amount of adsorbed MO dye onto LDH/CS1 and LDH/CS2 at the adsorption equilibriums. The isotherm curves for the LDH/CS1 and LDH/CS2 composite’s adsorption of MO are shown in Fig. 9. In this investigation, two well-known models, the Freundlich and Langmuir isotherms were employed to explain the interaction of adsorbate molecules with the adsorbent surface. The fundamental theory of the Langmuir model is that a monolayer forms on the adsorbent’s surface, indicating that just a single dye molecule can bind to one adsorption site and that intermolecular forces weaken with increasing distance. Additionally, the surface of the adsorbent is presumed to be homogenous, and its adsorption sites are equal and energetically equivalent^68,69^. This is displayed in Eq. (11). Otherwise, the Freundlich model describes the multilayer adsorption and the interaction of the molecules that have been adsorbed^70,71^. The Freundlich adsorption isotherm has the following nonlinear form Eq. (12).

11 $$q_{e} \, = \,q_{\max } \frac{{K_{L} C_{e} }}{{1 + K_{L} C_{e} }}$$

12 $$q_{e} \, = \,K_{f} .C_{e}^{{{\raise0.7ex\hbox{$1$} \!\mathord{\left/ {\vphantom {1 n}}\right.\kern-0pt} \!\lower0.7ex\hbox{$n$}}}}$$

whereas *q*_*e*_ (mg/g) represented the quantity of dye adsorbed at equilibrium, *q*_*max*_ (mg/g) represented the maximal adsorption capacity. *K*_*L*_ the Langmuir correlative adsorption constant. *K*_*F*_ is the Freundlich correlative adsorption constant, and the intensity of adsorption is denoted by n.

The separation factor or equilibrium parameter *R*_*L*_ is the other important parameter which can be determined as

13 $$R_{L} \, = \,\frac{1}{{1 + K_{L} C_{o} }}$$

Here, *K*_*L*_ is the Langmuir constant (1/mg) and C_o_ (mg/L) is the greatest dye concentration. The value of *R*_*L*_ indicates weather the isotherm is irreversible (*R*_*L*_ = 0), favorable (0 < 1), linear (*R*_*L*_ = 1), or unfavorable (*R*_*L*_ > 1). The observed *R*_*L*_ values for MO adsorption into LDH/CS1 and LDHCS2 ranged between 1 and 0, indicating a favorable adsorption^68^.

Since the two models' regression coefficient values (*R*^*2*^) are near to 1, the Freundlich and Langmuir isotherm models may both precisely assume the equilibrium data for the MO adsorption onto the LDH/CS1 and LDH/CS2. It’s interesting to note that rising MO concentrations cause dye to be adsorbed into LDH/CS (Fig. 9). From Table 3, the calculated value of n (> 1) in the Freundlich equations indicated a favorable adsorption process (n = 1.49 and 1.709 for LDH/CS1 and LDH/CS2, respectively). The Langmuir equilibrium constant *K*_*L*_ value of LDH/CS2 (0.0059 L/mg) was higher than that of LDH/CS1 (0.0034 L/mg). The greater the *K*_*L*_ value, the higher the energy sites and affinity of the adsorbent. Based on the values of *q*_*max*_ calculated by the Langmuir equation, the MO adsorption capacity for LDH/CS1 and LDH/CS2 was about 160.76 and 165.89 mg/g, respectively. Figure 9 and Table 3 demonstrate that the Langmuir adsorption capabilities of LDH/CS2 towards MO are higher than those of LDH/CS1. In addition, the Freundlich model's description of MO adsorption on LDH/CS2 is higher than that of LDH/CS1 (Table 3).

### Kinetic models

Kinetic models have been employed to study the mechanism of adsorption and potential rate-controlling processes so as to choose the best functional parameters for the complete batch process. The nonlinear pseudo-first order Eq. (14), pseudo-second-order Eq. (15), and intra-particle diffusion kinetic models Eq. (16) were examined in order to explore the kinetics of the adsorption of MO onto LDH/CS1 and LDH/CS2^71–73^. According to the pseudo-first-order kinetic model, the attraction occurs via the physical adsorption approach, whereas the pseudo second-order model is depicted based on chemisorption^74–76^. In addition to the intraparticle diffusion model, is employed to understand the diffusion mechanism^77^.

14 $$q_{t} \, = \,q_{e} (1 - e^{{ - K_{1} t}} ),$$

15 $$q_{t} = \frac{{q_{e}^{2} K_{2 } {\text{t}}}}{{1 + q_{e} { }K_{2} {\text{t}}}},$$

16 $$q_{t} = K_{ip} \sqrt t + C_{ip} .$$

The adsorption quantities at time *t* and equilibrium are denoted by *q*_*t*_ and *q*_e_ (mg/g), respectively. The rate constants of the pseudo-first-order, pseudo-second-order, and intra-particle diffusion kinetic models are represented by *K*_1_ (min^−1^), *K*_2_ (g/mg.min), and *K*_ip_ (mg/g min^1/2^), respectively. *C*_*ip*_ is the adsorption constant.

**Figure 10:**
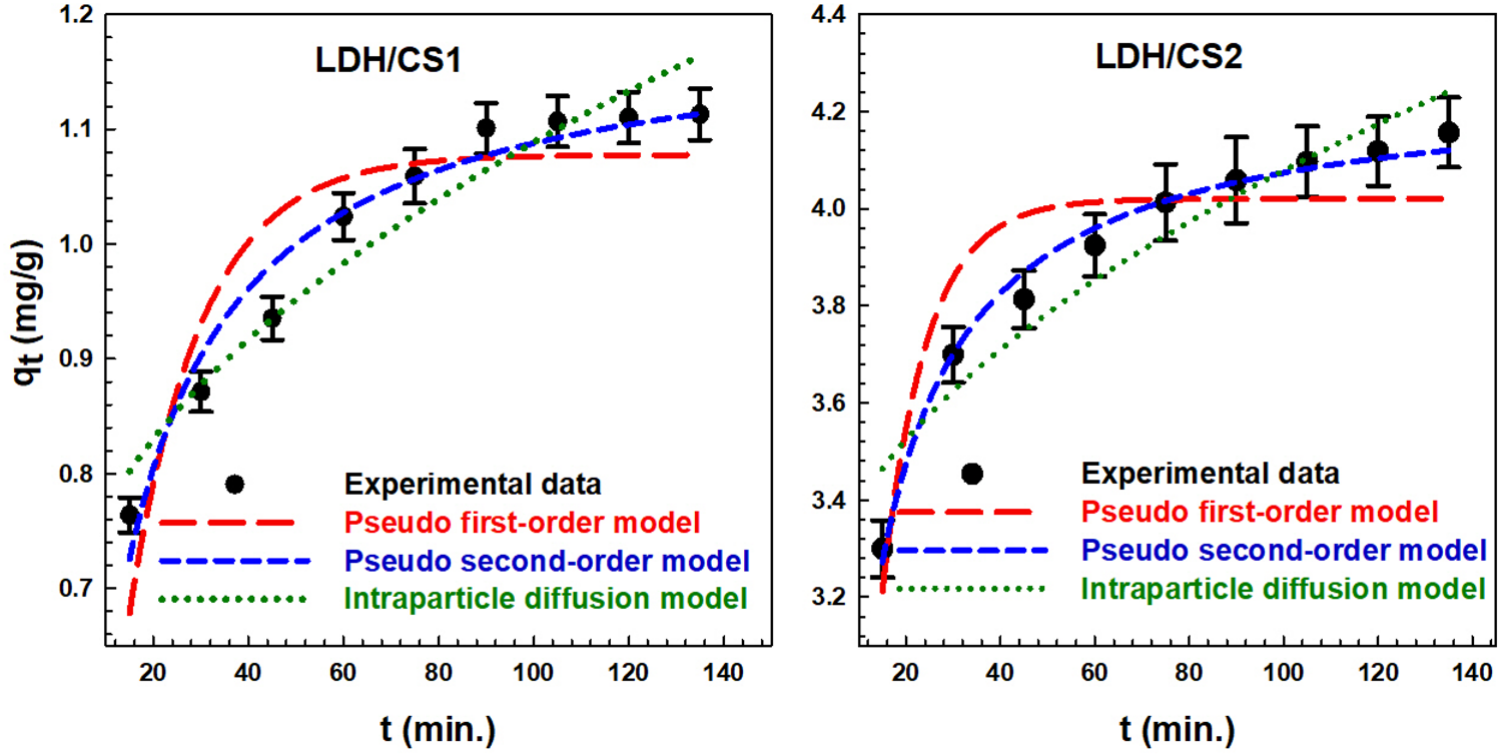
Adsorption kinetics of MO on LDH/CS1 and LDH/CS2 samples.

Figure 10 shows the fitted lines for the pseudo-first-order, pseudo-second-order, and intra-particle diffusion models. Table 4 displays a list of the relevant parameters and correlation coefficients. The model that is the best fit for illustrating the suitable adsorption process is determined using the regression coefficient (*R*^*2*^). Compared to the intra-particle diffusion and pseudo-first-order model, the *R*^*2*^ values in the pseudo-second-order model are greater and more closely associated with one. According to the experimental findings, the MO adsorption processes on LDH/CS1 and LDH/CS2 were chemical adsorptions involving electron transfer or valence force sharing^78^. The values of *q*_*cal*_ for LDH/CS1 and LDH/CS2 were 1.193 and 4.258 mg/g, respectively, indicating that LDH/CS2 is greater than LDH/CS1 in terms of MO adsorption capacity. Table 5 shows the comparison findings for calculated adsorption capacity and other parameters^79–81^.

**Table 4:** The adsorption kinetic models and their parameters obtained from the fitting results.

Models	Parameters	LDH/CS1	LDH/CS2
Pseudo-first-order	*q*_*e* cal_ (mg/g)	1.078 ± 0.004	4.020 ± 0.002
	*K*_*1*_ (min^−1^)	0.066	0.107
	*R*^*2*^	0.901	0.907
	SSE	0.007	0.018
	MPSD (%)	0.037	0.040
	∆q	0.44	0.467
Pseudo-second-order	*q*_*e* cal_ (mg/g)	1.193 ± 0.003	4.258 ± 0.001
	*K*_*2*_ (g/mg.min)	0.086	0.051
	*R*^*2*^	0.980	0.994
	SSE	0.006	0.010
	MPSD (%)	0.019	0.020
	∆q	1.026	0.350
Intra-particle diffusion	*K*_*ip*_ (mg/g min^1/2^)	0.046	0.100
	*C*_*ip*_ (mg/g)	0.621	3.076
	*R*^*2*^	0.960	0.950

**Table 5:** Reported competitive adsorption capacities of some adsorbents for different pollutants.

Adsorbents	Adsorbate	Pollutants concentration used (mg/L)	Amount of adsorbent	Adsorption capacity (mg/g)	Ref
Zn–Co–Fe/LDH	Methylene blue	100	0.1 g	58.26	^40^
Ti − Fe/LDH-chitosan	Cadmium Phosphate Benzoquinone	25 20 20	0.05 mg/L	250 50 69.4	^79^
MgAl-LDH/chitosan	As (V) ions	10.6	1.5 g/L	69.29	^80^
ZnO/chitosan	Ethyl orange	1.0	1 g of chitosan and 2 wt% of zinc oxide	0.014	^81^
LDH/CS1	MO	20	0.1 g	160.78	This work
LDH/CS2	MO	20	0.1 g	165.89	

### Statistical analyses

Statistical analyses of the kinetic data were fitted with different models by nonlinear regression. A minimization procedure has been adopted to solve kinetic equations by minimizing the sum of error squared (SSE) between the predicted values and the experimental data using Eq. (17)^82^. Marquardt’s percent standard deviation (MPSD), although used in adsorption kinetics analysis, is an error function that is a modification of the geometric mean distribution Eq. (18). It is based on the number of degrees of freedom of a system^83,84^. Meanwhile, the hybrid fractional error function (∆q) is used to improve the applicability of SSE at a lower concentration. The error function is divided by the measured value Eq. (19)^83,84^.

17 $${\text{SSE}}\, = \,\mathop \sum \limits_{i = 1}^{n} \left( {q_{cal} - q_{exp} } \right)^{2}_{i}$$

18 $${\text{MPSD}}\, = \,\left[ {\surd \frac{1}{n - p}\mathop \sum \limits_{i = 1}^{n} \left( {\frac{{q_{exp} - q_{cal} }}{{q_{exp} }}} \right)^{2}_{i} } \right]100$$

19 $$\Delta q \, = \,\frac{100}{{n - p}}\mathop \sum \limits_{i = 1}^{p} \left( {\frac{{q_{exp} - q_{cal} }}{{q_{exp} }}} \right)_{i}$$

where *q*_*cal*_ is the calculated amount of MO adsorbed, *q*_exp_ is the experimental amount of MO adsorbed, *n* is the data points, and p is the number of parameters in the model. The sum of error squared (SSE), Marquardt’s percent standard deviation (MPSD), and hybrid fractional error function (∆q) based on the actual deviation between the experimental points and predicted values are given in Table 4. From Table 4 the pseudo-second order equation provides better fitting to the experimental data points than other kinetics models.

### Impact of temperature on thermodynamic parameters

**Figure 11:**
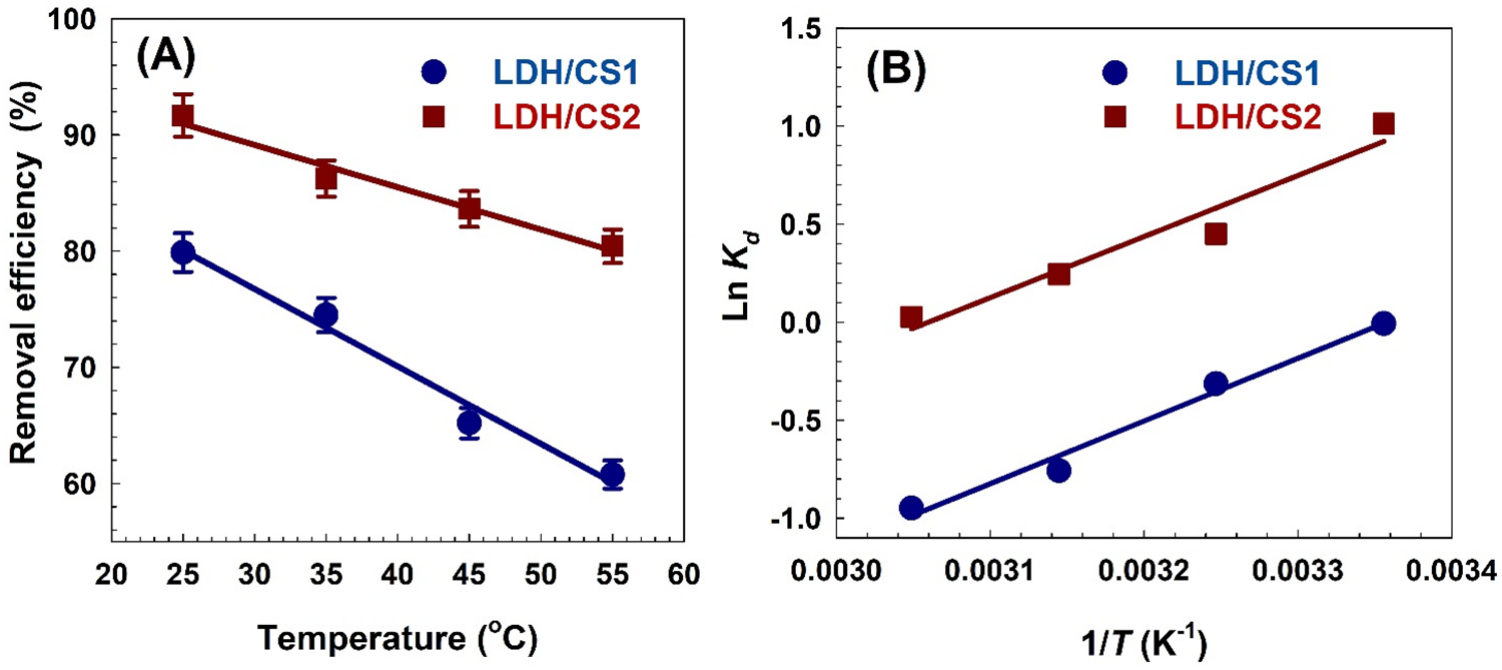
(**A**) The removal efficiency as a function of temperature on the adsorption of MO onto the LDH/CS1 and LDH/CS2 adsorbents and (**B**) The plot of Ln *K*_*d*_ vs 1/*T* which is used to calculate the thermodynamic parameters.

Adsorption process is significantly impacted by temperature. The MO adsorption onto LDH/CS1 and LDH/CS2 adsorbents was investigated with temperatures ranging from 298 to 328 K under optimal conditions to evaluate the influence of temperature. Figure 11A illustrates how temperature affects MO’s ability to bind to the LDH/CS1 and LDH/CS2 adsorbents. It was discovered that, as the solution temperature rose, MO’s adsorption on LDH/CS1 and LDH/CS2 reduced. This is explained by the adsorption process’s exothermic spontaneity as well as the fact that at high temperatures, interactions between dye molecules and adsorbent active sites become weaker^85^. The thermodynamic parameters standard enthalpy change (ΔH^o^), Gibbs free energy change (ΔG^o^), and standard entropy change (ΔS^o^) were examined in order to gain a better understanding of how temperature impacts adsorption. Tests were conducted using 0.1 g of the adsorbents LDH/CS1 and LDH/CS2, and 20 mg/L of MO solutions at 25, 35, 45, and 55 °C. The fitted thermodynamics are shown in Fig. 11B, and the predicted thermodynamic parameter values are given in Table 6. The thermodynamic parameters ΔG^o^, ΔH^o^, and ΔS^o^ were computed^86,87^ using the following equations.

20 $$\ln K_{d} = \frac{{\Delta S^{o} }}{R} - \frac{{\Delta H^{o} }}{RT}$$

21 $$\Delta {\text{G}}^{{\text{o}}} = - RT\ln K_{d}$$

where *R* represents the universal gas constant (8.314 J/mole K), *T* is the absolute temperature (K), and *K*_*d*_ = *q*_*e*_/*C*_*e*_ is the thermodynamic equilibrium constant (L/g). The slope and intercept of the plot of Ln *K*_*d*_ vs 1/*T* were used to calculate ΔH^o^ and ΔS^o^, respectively.

**Table 6:** Thermodynamic parameters for adsorption.

Adsorbent	*∆H*^*o*^ (kJ/mol)	*∆S*^*o*^ (J/(mol.K))	*∆G*^*o*^ (kJ/mol)				*R*^*2*^
			25 °C	35 °C	45 °C	55 °C	
LDH/CS1	− 26.59	− 89.24	− 0.01	− 0.80	− 2.00	− 2.58	0.98
LDH/CS2	− 25.88	− 79.20	2.51	1.15	0.64	0.07	0.95

The negative values of ∆H^o^ (− 26.59 and − 25.88 kJ/mol for LDH/CS1 and LDH/CS2, respectively) reflect the adsorption process exothermic nature, and the negative values of ∆S^o^ (− 89.24 and − 79.20 J/(mol.K) for LDH/CS1 and LDH/CS2, respectively) reflect a decrease in randomness at the solid/solution interactions in the adsorption procedure^88^. The typical Gibbs free energy change (∆G^o^) values (ranging from − 0.01: − 2.58 kJ/mol for 25–55 °C) were negative at several temperatures on the MO adsorption into LDH/CS1, indicating the spontaneity and feasibility of the adsorption process^89^. With rising temperatures, the process became more spontaneous^90^. The ∆G^o^ values (ranging from 2.51: 0.073 kJ/mol for 25–55 °C) were positive at several temperatures on the adsorption of MO into LDH/CS2, indicating that the process of the adsorption is nonspontaneous. The exothermic nature of the reaction may be the reason for the rise in ∆G^o^ values with increasing temperature^91^.

### Adsorption mechanism

**Figure 12:**
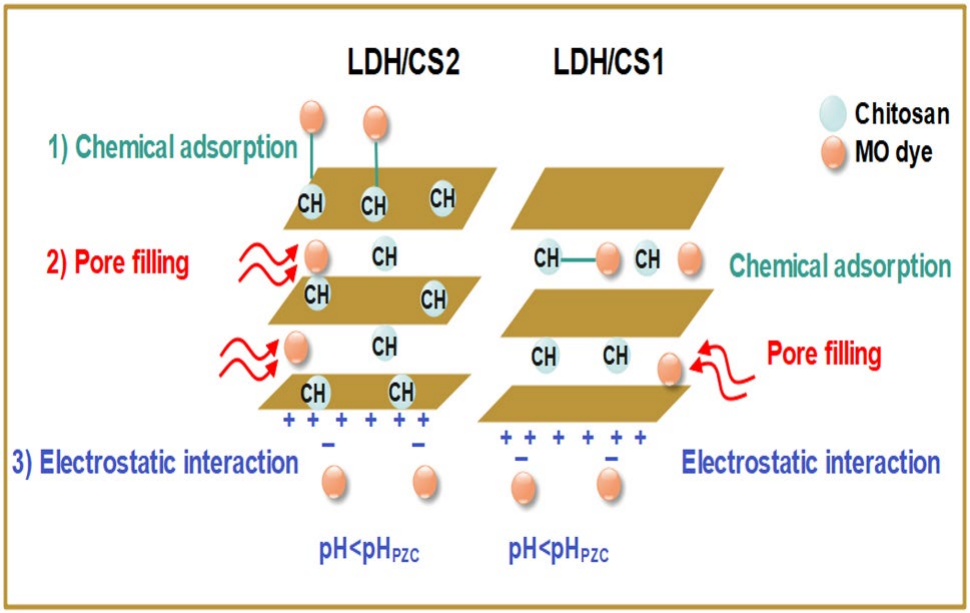
Possible adsorption mechanisms between LDH/CS and the MO system.

The adsorption mechanism is critical to understanding the process that occurs between adsorbent and adsorbate. Adsorption isotherms, adsorption kinetics, and adsorption characterization are essential requirements for building adsorption systems, and they can give useful information about reaction processes. In addition, the pH of the solution may influence the complexation behavior of the functional groups in LDH/CS and MO. LDH/CS2 showed greater adsorption performance for MO than LDH/CS1. Figure 12 illustrates the interaction mechanisms between LDH/CS and the MO system. The presence of functional groups on the surface of LDH/chitosan composites, such as hydroxyl (–OH), amino (–NH_2_), and carboxyl (–COOH) groups, can facilitate dye adsorption via hydrogen bonding and chemical complexation. In addition, the fitting parameters indicate that the pseudo-second-order model (R^2^ = 0.980 and 0.994 for LDH/CS1 and LDH/CS2, respectively) can well fit the kinetic processes at the LDH/CS confirming the chemical adsorptions. Furthermore, the electrostatic attraction mechanism is described as the interaction between the anionic MO and positively charged sites on the surface of LDH/CS when the pH solution < pH_PZC_ (pH solution < 7.2 and 6.6 for LDH/CS1 and LDH/CS2, respectively). Moreover, the fitting parameters of the pore filling mechanism indicate that the kinetic processes at the LDH/CS can be adequately suited by the intra-particle diffusion model (R^2^ = 0.960 and 0.950 for LDH/CS1 and LDH/CS2, respectively).

## Conclusion

In order to remove MO from an aqueous solution, LDH/CS1 and LDH/CS2 were produced in this investigation using a hydrothermal and coprecipitation approach. The process of adsorption was performed at varied contact times, pH values, dye concentrations, and LDH doses. The morphology of LDHs was examined using SEM images, which revealed plate-like particles that were stacked on top of one another in LDH/CS1 and tended to clump together in LDH/CS2. As analyzed from the PAL results, the increasing value of *I*_*3*_ in LDH/CS2 suggests that the number of holes increased but the size of each hole decreased. The increasing value of *I*_*3*_ in LDH/CS2 indicates that the number density of pores increased, which led to a decrease in the value of τ_3_ and hence the hole radius and V compared to the value in LDH/CS2. Two isothermal models were used to study equilibrium adsorption. The Langmuir and Freundlich models can clearly agree with the isotherm methods at the LDH/CS1 and LDH/CS2 sites according to the fitting parameters, where the *q*_*max*_ was determined to be 160.78 and 165.89 mg/g, respectively. A pseudo second-order model was used to evaluate the kinetics of LDH adsorption in two samples. At room temperature, the removal efficiency of MO was 72.8% for LDH/CS1 and 91.7% for LDH/CS2. Temperature studies were carried out at 25, 35, 45, and 55 °C to investigate the thermodynamic parameters ∆H^o^, ∆S^o^, and ∆G^o^. The calculated values show exothermic and non-spontaneous LDH/CS1 adsorption mechanisms. The values of ∆G^o^ in the adsorption of MO in LDH/CS2 were positive at different temperatures, indicating that the adsorption process is not spontaneous. Based on earlier investigations, it is possible to conclude that LDH/CS1 and LDH/CS2 can be used as adsorbents to remove a variety of other contaminants found in wastewater, including heavy metals, organic pollutants, and emerging contaminants like pharmaceuticals. LDH/CS1 and LDH/CS2 also have potential applications beyond the scope of the current study for a lot of applications other than water treatment, such as catalysis, drug delivery, or gas separation, by employing their unique structural and functional features.

## Data Availability

The datasets used and/or analysed during the current study available from the corresponding author on reasonable request.

## References

[1] Islam T, Repon MR, Islam T, Sarwar Z, Rahman MM (2023). Impact of textile dyes on health and ecosystem: A review of structure, causes, and potential solutions. Environ. Sci. Pollut. Res..

[2] Sadiq AC, Rahim NY, Suah FBM (2020). Adsorption and desorption of malachite green by using chitosan-deep eutectic solvents beads. Int. J. Biol. Macromol..

[3] Mohadi R, Siregar PMSBN, Palapa NR, Lesbani A (2022). Preparation of Zn/Al-chitosan composite for the selective adsorption of methylene blue dye in water. Makara J. Sci..

[4] Hanafi MF, Sapawe N (2020). A review on the water problem associate with organic pollutants derived from phenol, methyl orange, and remazol brilliant blue dyes. Mater. Today Proc..

[5] Liu M (2017). High efficient removal of dyes from aqueous solution through nanofiltration using diethanolamine-modified polyamide thin-film composite membrane. Sep. Purif. Technol..

[6] Cai M (2023). Formation and stabilization of NiOOH by introducing α-FeOOH in LDH: Composite electrocatalyst for oxygen evolution and urea oxidation reactions. Adv. Mater..

[7] Boucenna N, Mokhtari-Belkhadem F, Bouteiba A, Sahel K, Medina F, Lounis M (2023). Catalytic ozonation of n-methyldiethanolamine over mixed oxides derived from Mg/Fe-LDH. Water Sci. Technol..

[8] Nia SB, Pooresmaeil M, Namazi H (2020). Carboxymethylcellulose/layered double hydroxides bio-nanocomposite hydrogel: A controlled amoxicillin nanocarrier for colonic bacterial infections treatment. Int. J. Biol. Macromol..

[9] Fang D (2022). New insights into the arrangement pattern of layered double hydroxide nanosheets and their ion-exchange behavior with phosphate. Chem. Eng. J..

[10] Huang Z, Wang T, Shen M, Huang Z, Chong Y, Cui L (2019). Coagulation treatment of swine wastewater by the method of in-situ forming layered double hydroxides and sludge recycling for preparation of biochar composite catalyst. Chem. Eng. J..

[11] Bessaies H (2020). Synthesis of novel adsorbent by intercalation of biopolymer in LDH for the removal of arsenic from synthetic and natural water. J. Environ. Sci..

[12] Bagherzadeh M, Salehi G, Rabiee N (2024). Rapid and efficient removal of methylene blue dye from aqueous solutions using extract-modified Zn–Al LDH. Chemosphere.

[13] Zaghloul A, Benhiti R, Soudani A, Chiban M, Zerbet M, Sinan F (2019). Removal of methyl orange from aqueous solution using synthetic clay type MgAl-LDH: Characterization, isotherm and thermodynamic studies. Mediterr. J. Chem..

[14] Potgieter JH, Pardesi C, Pearson S (2021). A kinetic and thermodynamic investigation into the removal of methyl orange from wastewater utilizing fly ash in different process configurations. Environ. Geochem. Health.

[15] Sonu K, Sogani M, Syed Z, Rajvanshi J, Sengupta N (2022). Effectiveness of rice husk in the removal of methyl orange dye in constructed wetland-microbial fuel cell. Bioresour. Technol. Reports..

[16] Radoor S, Karayil J, Jayakumar A, Parameswaranpillai J, Siengchin S (2021). Efficient removal of methyl orange from aqueous solution using mesoporous ZSM-5 zeolite: Synthesis, kinetics and isotherm studies. Colloids Surf. A Physicochem. Eng. Asp..

[17] Tsai F-C (2014). Adsorptive removal of methyl orange from aqueous solution with crosslinking chitosan microspheres. J. Water Process Eng..

[18] AbdelGhany GS, Ebrahiem EE, Mohamed HFM, Ali GAM, Shehata N (2021). Eco-friendly activated carbon developed from rice hulls for chromium and iron ion removal. J. Environ. Eng. Sci..

[19] Pal J, Deb MK, Deshmukh DK, Verma D (2013). Removal of methyl orange by activated carbon modified by silver nanoparticles. Appl. Water Sci..

[20] Sejie FP, Nadiye-Tabbiruka MS (2016). Removal of methyl orange (MO) from water by adsorption onto modified local clay (kaolinite). Phys. Chem..

[21] Monash P, Pugazhenthi G (2014). Utilization of calcined Ni-Al layered double hydroxide (LDH) as an adsorbent for removal of methyl orange dye from aqueous solution. Environ. Prog. Sustain. Energy.

[22] Woo MA, Kim TW, Paek M-J, Ha H-W, Choy J-H, Hwang S-J (2011). Phosphate-intercalated Ca–Fe-layered double hydroxides: Crystal structure, bonding character, and release kinetics of phosphate. J. Solid State Chem..

[23] Sayed H, Mahmoud R, Mohamed HFM, Gaber Y, Shehata N (2022). Co and Ni double substituted Zn–Fe layered double hydroxide as 2d nano-adsorbent for wastewater treatment. Key Eng. Mater..

[24] Mahmoud R, Mohamed HFM, Hafez SHM, Gadelhak YM, Abdel-Hady EE (2022). Valorization of spent double substituted Co–Ni–Zn–Fe LDH wastewater nanoadsorbent as methanol electro-oxidation catalyst. Sci. Rep..

[25] Daud M (2019). A review on the recent advances, challenges and future aspect of layered double hydroxides (LDH)—Containing hybrids as promising adsorbents for dyes removal. J. Mol. Liq..

[26] Yang Z (2016). Utilization of LDH-based materials as potential adsorbents and photocatalysts for the decontamination of dyes wastewater: A review. RSC Adv..

[27] Missau J, Bertuol DA, Tanabe EH (2021). Highly efficient adsorbent for removal of crystal violet dye from aqueous solution by CaAl/LDH supported on biochar. Appl. Clay Sci..

[28] Hafez SHM, Mohamed HFM, Abdel-Hady EE (2023). Catalyzing innovation of exploring the vast potential of low-cost alternative adsorbents in diverse applications: A review. Microchem. J..

[29] GadelHak Y, Hafez SHM, Mohamed HFM, Abdel-Hady EE, Mahmoud R (2023). Nanomaterials-modified disposable electrodes and portable electrochemical systems for heavy metals detection in wastewater streams: A review. Microchem. J..

[30] Annadurai G, Ling LY, Lee J-F (2008). Adsorption of reactive dye from an aqueous solution by chitosan: Isotherm, kinetic and thermodynamic analysis. J. Hazard. Mater..

[31] Dotto GL, Pinto LAA (2011). Adsorption of food dyes acid blue 9 and food yellow 3 onto chitosan: Stirring rate effect in kinetics and mechanism. J. Hazard. Mater..

[32] Kyzas GZ, Lazaridis NK (2009). Reactive and basic dyes removal by sorption onto chitosan derivatives. J. Colloid Interface Sci..

[33] Wong YC, Szeto YS, Cheung Wh, McKay G (2004). Adsorption of acid dyes on chitosan—Equilibrium isotherm analyses. Process Biochem..

[34] Mahmoodi NM, Salehi R, Arami M, Bahrami H (2011). Dye removal from colored textile wastewater using chitosan in binary systems. Desalination.

[35] Son BC, Park K, Song SH, Yoo YJ (2004). Selective biosorption of mixed heavy metal ions using polysaccharides. Korean J. Chem. Eng..

[36] Boddu VM, Abburi K, Talbott JL, Smith ED, Haasch R (2008). Removal of arsenic (III) and arsenic (V) from aqueous medium using chitosan-coated biosorbent. Water Res..

[37] Mohammed WM, Awad S, Abdel-Hady EE, Mohamed HFM, Elsharkawy YS, Elsharkawy MRM (2023). Nanostructure analysis and dielectric properties of PVA/sPTA proton exchange membrane for fuel cell applications: Positron lifetime study. Radiat. Phys. Chem..

[38] Gomaa MM, Hugenschmidt C, Dickmann M, Abdel-Hady EE, Mohamed HFM, Abdel-Hamed MO (2018). Crosslinked PVA/SSA proton exchange membranes: Correlation between physiochemical properties and free volume determined by positron annihilation spectroscopy. Phys. Chem. Chem. Phys..

[39] Irawan C, Ramadhan MW, Nata IF, Putra MD (2020). The treatment of raw water sources of drinking water using chitosan/Mg/Al–LDH composites: Problem cases in municipal waterworks in Banjarmasin. IOP Conf. Ser. Earth Environ. Sci..

[40] Abdel-Hady EE, Mahmoud R, Hafez SHM, Mohamed HFM (2022). Hierarchical ternary ZnCoFe layered double hydroxide as efficient adsorbent and catalyst for methanol electrooxidation. J. Mater. Res. Technol..

[41] Billah REK (2023). Multifunctional cross-linked shrimp waste-derived chitosan/MgAl-LDH composite for removal of As (V) from wastewater and antibacterial activity. ACS Omega.

[42] Mohamed HFM, Abd El-Aziz NS (2001). Study on polystyrene via positron annihilation lifetime and Doppler broadened techniques. Polymer.

[43] Olsen JV, Kirkegaard P, Pedersen NJ, Eldrup M (2007). PALSfit: A new program for the evaluation of positron lifetime spectra. Phys. Status Solidi C..

[44] Azzam EMS, Solyman SM, Abd-Elaal AA (2016). Fabrication of chitosan/Ag-nanoparticles/clay nanocomposites for catalytic control on oxidative polymerization of aniline. Colloids Surf. A Physicochem. Eng. Asp..

[45] Mohamed HFM, El-Sayed A, Hussien AZ (2001). On irradiated poly (ethylene naphthalate) studied by positron annihilation lifetime spectroscopy. Mater. Sci. Forum.

[46] Kamal W, El Rouby WMA, El-Gendy AO, Farghali AA (2018). Bimodal applications of LDH-chitosan nanocomposite: Water treatment and antimicrobial activity. IOP Conference Series: Materials Science and Engineering.

[47] Kumar MSC, Selvam V, Vadivel M (2012). Synthesis and characterization of silane modified iron (III) oxide nanoparticles reinforced chitosan nanocomposites. Eng. Sci. Adv. Tech..

[48] Nejati K, Keypour H, Nezhad PDK, Rezvani Z, Asadpour-Zeynali K (2015). Preparation and characterization of cetirizine intercalated layered double hydroxide and chitosan nanocomposites. J. Taiwan Inst. Chem. Eng..

[49] Dtie, UNEP. Converting Waste Agricultural Biomass into a Resource. Compendium of Technologies. Osaka, United Nations Environment Programme. 1–437 (2009).

[50] Ţălu Ş, Janus K, Stach S (2017). Nanoscale patterns in carbon–nickel nanocomposite thin films investigated by AFM and stereometric analysis. Automot. Ind..

[51] Ţălu Ş, Shcherbinin DP, Konshina EA, Gladskikh IA (2021). Stereometric and fractal analysis of granulated silver films used in thin-film hybrid structures. J. Microsc..

[52] Dalla Nora FB (2020). Adsorptive potential of Zn–Al and Mg–Fe layered double hydroxides for the removal of 2–nitrophenol from aqueous solutions. J. Environ. Chem. Eng..

[53] Kong M (2011). Tuning the relative concentration ratio of bulk defects to surface defects in TiO_2_ nanocrystals leads to high photocatalytic efficiency. J. Am. Chem. Soc..

[54] Sun W, Li Y, Shi W, Zhao X, Fang P (2011). Formation of AgI/TiO 2 nanocomposite leads to excellent thermochromic reversibility and photostability. J. Mater. Chem..

[55] Jean JYC, Mallon PE, Schrader DM (2003). Principles and Applications of Positron and Positronium Chemistry.

[56] Dlubek G (2003). Ortho-positronium lifetime distribution analyzed with MELT and LT and free volume in poly (ε-caprolactone) during glass transition, melting, and crystallization. J. Polym. Sci. Part B Polym. Phys..

[57] Giebel D, Kansy J (2010). A new version of LT program for positron lifetime spectra analysis. Mater. Sci. Forum.

[58] Kansy J (1996). Microcomputer program for analysis of positron annihilation lifetime spectra. Nucl. Instrum. Methods Phys. Res. Sect. A Accel. Spectrom. Detect. Assoc. Equip..

[59] Nakanishi H (1988). Microscopic surface tension studied by positron annihilation. International Symposium on Positron Annihilation Studies of Fluids.

[60] Tao SJ (1972). Positronium annihilation in molecular substances. J. Chem. Phys..

[61] Eldrup M, Lightbody D, Sherwood JN (1981). The temperature dependence of positron lifetimes in solid pivalic acid. Chem. Phys..

[62] Kamel MSA, Mohamed HFM, Abdel-Hamed MO, Abdel-Hady EE (2019). Characterization and evaluation of Nafion HP JP as proton exchange membrane: Transport properties, nanostructure, morphology, and cell performance. J. Solid State Electrochem..

[63] Kobayashi Y (2016). Hole size distributions in cardo-based polymer membranes deduced from the lifetimes of ortho-positronium. J. Phys. Conf. Ser..

[64] Mohamed HFM, Owais A (2018). Tracking free volume changes in bisphenol-a based polycarbonate sheets after treatment with liquid acetone. J. Polym. Res..

[65] Iftekhar S, Srivastava V, Ramasamy DL, Naseer WA, Sillanpää M (2018). A novel approach for synthesis of exfoliated biopolymeric-LDH hybrid nanocomposites via in-stiu coprecipitation with gum Arabic: Application towards REEs recovery. Chem. Eng. J..

[66] Bessaies H (2021). Characterization and physicochemical aspects of novel cellulose-based layered double hydroxide nanocomposite for removal of antimony and fluoride from aqueous solution. J. Environ. Sci..

[67] Abdel-Hady EE (2023). Textural properties and adsorption behavior of Zn–Mg–Al layered double hydroxide upon crystal violet dye removal as a low cost, effective, and recyclable adsorbent. Sci. Rep..

[68] Hall KR, Eagleton LC, Acrivos A, Vermeulen T (1966). Pore-and solid-diffusion kinetics in fixed-bed adsorption under constant-pattern conditions. Ind. Eng. Chem. Fundam..

[69] Langmuir I (1918). The adsorption of gases on plane surfaces of glass, mica and platinum. J. Am. Chem. Soc..

[70] Kang D (2013). Performance and mechanism of Mg/Fe layered double hydroxides for fluoride and arsenate removal from aqueous solution. Chem. Eng. J..

[71] Sepehr MN, Al-Musawi TJ, Ghahramani E, Kazemian H, Zarrabi M (2017). Adsorption performance of magnesium/aluminum layered double hydroxide nanoparticles for metronidazole from aqueous solution. Arab. J. Chem..

[72] Chen H, Zhao J, Wu J, Dai G (2011). Isotherm, thermodynamic, kinetics and adsorption mechanism studies of methyl orange by surfactant modified silkworm exuviae. J. Hazard. Mater..

[73] Chen L (2016). Hollow LDH nanowires as excellent adsorbents for organic dye. J. Alloys Compd..

[74] Wang J, Guo X (2020). Adsorption kinetic models: Physical meanings, applications, and solving methods. J. Hazard. Mater..

[75] Supriyadi D, Farhani AC, Sanjaya A, Soraya F (2019). Evaluation of kinetics adsorption models from Lampung ethnic textile industry wastewater for removal chromium onto modified activated sludge and zeolite adsorbent. IOP Conf. Ser. Earth Environ. Sci..

[76] Iftekhar S, Srivastava V, Wasayh MA, Hezarjaribi M, Sillanpää M (2020). Incorporation of inorganic matrices through different routes to enhance the adsorptive properties of xanthan via adsorption and membrane separation for selective REEs recovery. Chem. Eng. J..

[77] Oladoja NA (2016). A critical review of the applicability of Avrami fractional kinetic equation in adsorption-based water treatment studies. Desalin. Water Treat..

[78] Arshadi M, Mousavinia F, Amiri MJ, Faraji AR (2016). Adsorption of methyl orange and salicylic acid on a nano-transition metal composite: Kinetics, thermodynamic and electrochemical studies. J. Colloid Interface Sci..

[79] Mahmoud R, Moaty SA, Mohamed F, Farghali A (2017). Comparative study of single and multiple pollutants system using Ti-Fe chitosan LDH adsorbent with high performance in wastewater treatment. J. Chem. Eng. Data.

[80] Billah REK (2023). Multifunctional cross-linked shrimp waste-derived chitosan/MgAl-LDH composite for removal of As(V) from wastewater and antibacterial activity. ACS Omega.

[81] Ngadi N, Mahmud MA, Jusoh M, Rahman RA, Alias H (2014). Removal of ethyl orange dye using hybrid chitosan and zinc oxide. J. Teknol..

[82] Günay A, Arslankaya E, Tosun I (2007). Lead removal from aqueous solution by natural and pretreated clinoptilolite: adsorption equilibrium and kinetics. J. Hazard. Mater..

[83] William Kajjumba G, Emik S, Öngen A, Kurtulus Özcan H, Aydın S (2018). Modelling of adsorption kinetic processes—errors, theory and application. Advanced Sorption Process Applications.

[84] Demirbas E, Kobya M, Konukman AES (2008). Error analysis of equilibrium studies for the almond shell activated carbon adsorption of Cr (VI) from aqueous solutions. J. Hazard. Mater..

[85] Sabriye Y, Sema E (2011). Adsorption characterization of strontium on PAN/zeolite composite adsorbent. World J. Nucl. Sci. Technol..

[86] Horsfall Jnr M, Spiff AI (2005). Effects of temperature on the sorption of Pb2^+^ and Cd2^+^ from aqueous solution by caladium bicolor (wild cocoyam) biomass. Electron. J. Biotechnol..

[87] Pourfaraj R, Fatemi SJ, Kazemi SY, Biparva P (2017). Synthesis of hexagonal mesoporous MgAl LDH nanoplatelets adsorbent for the effective adsorption of brilliant yellow. J. Colloid Interface Sci..

[88] Stoller M, Pulido JMO, Di Palma L, Ferez AM (2015). Membrane process enhancement of 2-phase and 3-phase olive mill wastewater treatment plants by photocatalysis with magnetic-core titanium dioxide nanoparticles. J. Ind. Eng. Chem..

[89] Zhang L, Hu P, Wang J, Liu Q, Huang R (2015). Adsorption of methyl orange (MO) by Zr (IV)-immobilized cross-linked chitosan/bentonite composite. Int. J. Biol. Macromol..

[90] Ahmad AL, Chan CY, Abd Shukor SR, Mashitah MD (2009). Adsorption kinetics and thermodynamics of β-carotene on silica-based adsorbent. Chem. Eng. J..

[91] Elwakeel KZ, Atia AA, Guibal E (2014). Fast removal of uranium from aqueous solutions using tetraethylenepentamine modified magnetic chitosan resin. Bioresour. Technol..

